# The nuclear bodies formed by histone demethylase KDM7A

**DOI:** 10.1007/s13238-020-00783-x

**Published:** 2020-09-16

**Authors:** Hui Ming, Qianfeng Wang, Yuwen Zhang, Luzhang Ji, Lu Cheng, Xiangru Huo, Zixiang Yan, Zhexiao Liu, Yongjun Dang, Bo Wen

**Affiliations:** 1 MOE Key Laboratory of Metabolism and Molecular Medicine and Department of Biochemistry and Molecular Biology, School of Basic Medical Sciences, and Institutes of Biomedical Sciences, Shanghai, 200032 China; 2 State Key Laboratory of Genetic Engineering, Collaborative Innovation Center of Genetics and Development, Fudan University, Shanghai, 200438 China

**Dear Editor,**

In the nucleus of higher eukaryotes, chromatin occupies only a small proportion of the nuclear space, while many proteins and RNAs segregate into membrane-less nuclear bodies (NBs). These NBs follow a stochastic or ordered assembly model and constantly exchange components with the surrounding nucleoplasm (Jain et al., 2016). Typical NBs include nucleoli, nuclear speckles, paraspeckles, PML bodies, Cajal bodies, polycomb bodies and Sam68 bodies, which play critical roles in various biological processes such as ribosome assembly, RNA processing, and protein modification. The dysfunction of nuclear bodies may cause diseases, such as cancer (Li et al., 2019).

It has been revealed that NBs involve in the stress response. Stress could lead to cell-cycle arrest, apoptosis or reorganization of the nuclear architecture. NBs respond quickly to stress, altering their components or structure to help the cell to recover from abnormal states. Nucleolus, as the largest nuclear body, is regarded as a central hub in coordinating cellular stress response by regulating ribosome subunit biogenesis and cell cycle progression. In response to stress, nucleolus can control the activity of the tumor suppressor protein p53 or crosstalk with Cajal bodies (Boulon et al., 2010). As a ROS sensor, PML can activate oxidative stress-responsive p53 targets during the PML body biogenesis, which is dependent on the PML RING tetramerization (Wang et al., 2018).

The lysine demethylase 7A (KDM7A) functions as a dual-specificity demethylase for histone H3 Lys 9 and Lys 27 dimethylation (H3K9me2 and H3K27me2), two repressive histone marks. Under long-term nutrient starvation, the expression level of KDM7A increased in cancer cells (Osawa et al., 2011) (Fig. 1A). We were asking whether the increase of KDM7A links to NBs under environmental stimuli. Indeed, the immunofluorescence signal of KDM7A gathered as the larger size foci after 48-hour serum deprivation in HeLa cells (Fig. 1B and 1C). Most of these KDM7A foci are different from known NBs such as nuclear speckles, paraspeckles, PML bodies, Cajal bodies, polycomb bodies, Sam68 bodies, and RNA polymerase II condensates (Figs. 1D, S1A and S1B). We thus named these nuclear KDM7A foci as K-bodies. Additionally, we tested other stresses: the high salt treatment, but not amino acids starvation, can also lead to enlarged KDM7A foci (Fig. S1C–F), which implied that KDM7A condensates may form when cells undergo intense stresses.

**Fig. 1:**
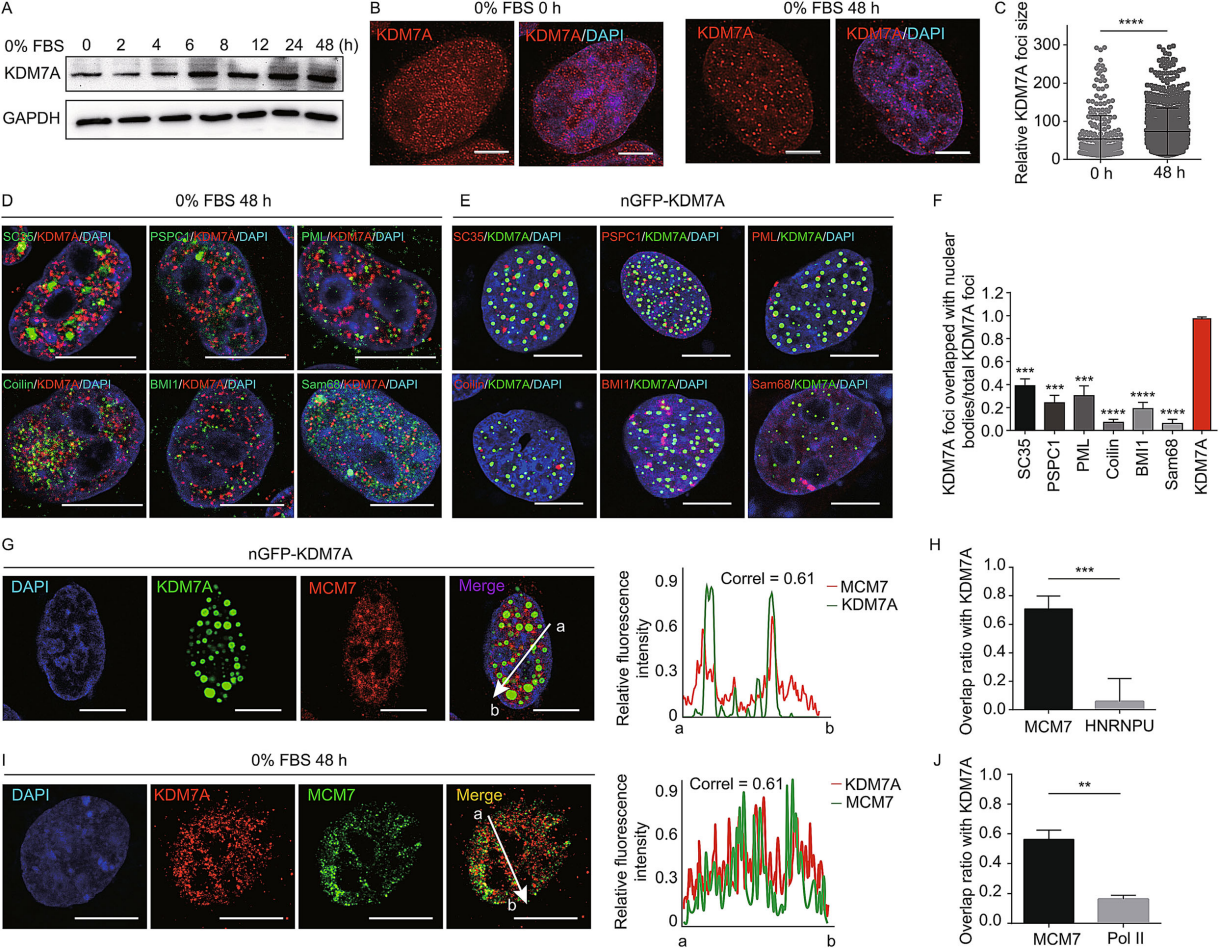

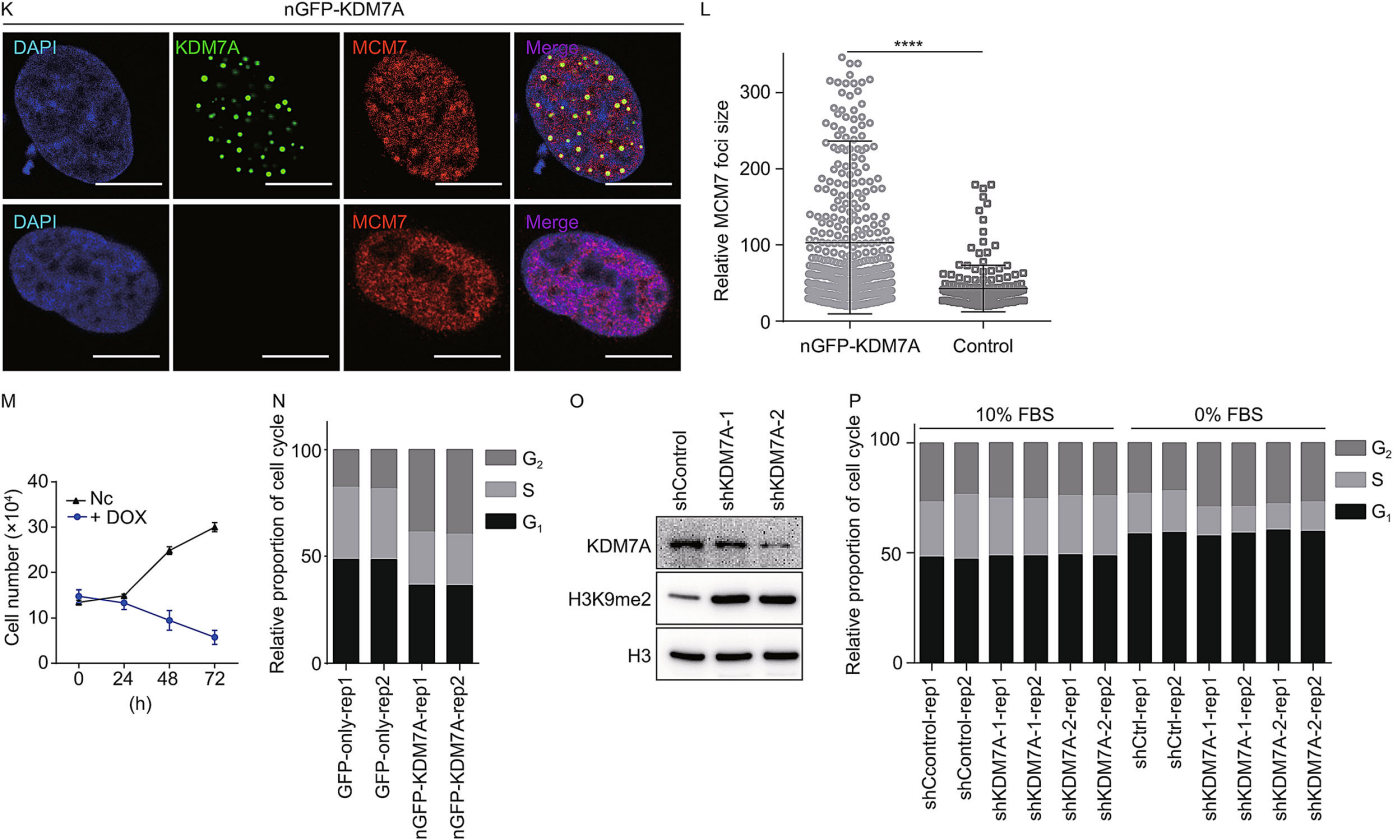
KDM7A marks nuclear bodies (K-bodies). (A) The expression level of KDM7A in HeLa cells under serum starvation condition as indicated by Western blot. GAPDH as loading control. (B) The location pattern of KDM7A in HeLa cells cultured in NC (DMEM with 10% FBS) and serum starvation (DMEM with 0% FBS) conditions. Scale bar, 10 µm. (C) The sizes of KDM7A foci in HeLa cells under normal and serum starvation condition. 7–8 cells were analyzed. ****P < 0.0001 by unpaired *t*-test. (D) Co-staining of KDM7A and SC35, PSPC1, PML, Coilin, BMI1 and Sam68 in HeLa cells treated with 0% FBS for 48 h, respectively. Scale bar, 10 µm. (E) U2OS cells transfected with nGFP-KDM7A were stained with SC35, PSPC1, PML, Coilin, BMI1 and Sam68. Scale bar, 10 µm. (F) The proportion of K-bodies overlapping with nuclear bodies compared to KDM7A antibody. 12-14 cells were analyzed. ***P < 0.001, ****P < 0.0001 by unpaired *t*-test. (G) The localization of KDM7A and MCM7 in U2OS cells transfected with nGFP-KDM7A. Scale bar, 10 µm. Right, relative fluorescence intensity of KDM7A and MCM7 from a to b. (H) The overlap ratio of MCM7 and HNRNPU with KDM7A condensates in nGFP-KDM7A overexpressed U2OS cells, respectively. 13–18 cells were analyzed. ***P < 0.001 by unpaired *t*-test. (I) The localization of MCM7 and KDM7A in HeLa cells treated with 0% FBS for 48 h. Scale bar, 10 µm. Right, relative fluorescence intensity of MCM7 and KDM7A from a to b. (J) The overlap ratio of MCM7 and Pol II with K-bodies in HeLa cells treated with 0% FBS for 48 h, respectively. 12–15 cells were analyzed. **P < 0.01 by unpaired *t*-test. (K) The localization of MCM7 in nGFP-KDM7A transfected U2OS cells and normal U2OS cells. Scale bar, 10 µm. (L) The relative sizes of MCM7 foci in U2OS cells transfected with nGFP-KDM7A and normal U2OS cells. 11–14 cells were analyzed. ****P < 0.0001 by unpaired *t*-test. (M) Cell number analysis of control and nGFP-KDM7A overexpressing U2OS cells with Dox-inducible system. (N) Cell cycle analysis of GFP-only and nGFP-KDM7A expressing cells by flow cytometry. (O) Western blot of KDM7A and H3K9me2 in shCtrl, shKDM7A-1 and shKDM7A-2 treated HeLa cells. H3 as the loading control. (P) Cell cycle analysis of HeLa cells cultured in normal or serum starvation condition treated with shCtrl, shKDM7A-1, shKDM7A-2 by flow cytometry

Furthermore, the expression of exogenous N-terminal tagged GFP-KDM7A (nGFP-KDM7A), which retains its histone demethylase activity (Fig. S2A), can also induce KDM7A foci (Fig. S2B). These nGFP-KDM7A foci can be clearly detected by immunofluorescence with the anti-KDM7A antibody, thereby validating the specificity of this antibody (Fig. S2C). Immunofluorescence imaging revealed that the nGFP-KDM7A foci are also largely independent of tested NBs (Figs. 1E, 1F and S2D).

To investigate the function of K-bodies, we analyzed KDM7A-associated proteins by performing immunoprecipitation (IP) experiments with GFP-trap in the U2OS cell line, which is a common cell model for NB studies (Fig. S3A). Through high-resolution liquid chromatography-tandem mass spectrometry (LC-MS), we identified 250 proteins that were associated with nGFP-KDM7A (Fig. S3B and Table S1). Based on Gene Ontology (GO) analysis, these proteins are related to biological processes such as rRNA process, DNA replication and DNA repair (Fig. S3C). Interestingly, the top GO term is rRNA process, probably because the abundant ribosomal proteins can be phases-separated, or the crosstalk occurs between nuclear bodies (Boulon et al., 2010). The top hit is minichromosome maintenance complex component 7 (MCM7), which is essential for the initiation of DNA replication (Pacek and Walter, 2004). Furthermore, we performed endogenous KDM7A IP and LC-MS experiments with serum-starved HeLa cells and identified 273 candidates (Table S2). Although these two proteome datasets conducted with different methods and cell models, there are 28 overlapped proteins between them, including cell cycle-related proteins such as MCM7, CDK1, RFC1, RFC2 and RFC5 (Fig. S3D). These data suggested that K-bodies induced by nutrient starvation and KDM7A condensates induced by overexpression may share some common components.

The co-localization of KDM7A and MCM7 was validated in nGFP-KDM7A transfected U2OS cells (Figs. 1G, 1H and S4A), and in serum-starved HeLa cells (Fig. 1I, 1J and S1B). MCM7 foci appear in nGFP-KDM7A overexpressed cells, but not in the un-transfected cells (Fig. 1K and 1L). Besides, upon KDM7A knockdown in serum-starved cells, the size of MCM7 foci decreased significantly (Fig. S4B and S4C), while the protein level of MCM7 was unchanged (Fig. S4D). These data indicate that KDM7A is required for the formation of MCM7 puncta. Furthermore, the induction of KDM7A condensates by nGFP-KDM7A expression in U2OS cells disrupted cell proliferation (Fig. 1M), and led to the altered cell cycle (Fig. 1N). Knockdown of KDM7A under the normal culture condition (10% FBS) didn’t cause a change in cell cycle. However, a serum starvation (0% FBS) shortened the S-phase, and knockdown of KDM7A made S-phase even shorter (Fig. 1O and 1P). These results implied that, under environmental stimuli, K-bodies could help cells to resist the stress and maintain certain cellular activity by sequestering proteins related to DNA replication and other processes.

Through the super-resolution microscopy, we can see that nGFP-KDM7A induced KDM7A condensates are hollow spheres. MCM7 could distribute inside or embed in the shell of different K-bodies (Fig. 2A). As KDM7A is a histone demethylase, we were prompted to investigate the relationship between chromatin and K-bodies. We transfected the nGFP-KDM7A vector into NIH3T3 cells whose chromatin is more condensed compared to U2OS. Immunofluorescence data showed that histone H3 was distributed around KDM7A condensates (Fig. S5A). Super-resolution fluorescence microscopy revealed that the H3 signals are wrapped around the “K-body ring” (Fig. 2B). Furthermore, we labeled KDM7A with 3, 3’-diaminobenzidine (DAB), which can be electron microscopy (EM)-visible after treatment with osmium tetroxide (Martell et al., 2012). Consistently, as revealed by EM imaging, DAB-labeled KDM7A forms a ring, which was wrapped by electron-dense chromatin (Fig. 2C). Collectively, the results of super-resolution fluorescence microscopy and TEM-based imaging indicate that chromatin is distributed around hollow, spherical KDM7A condensates.

**Fig. 2:**
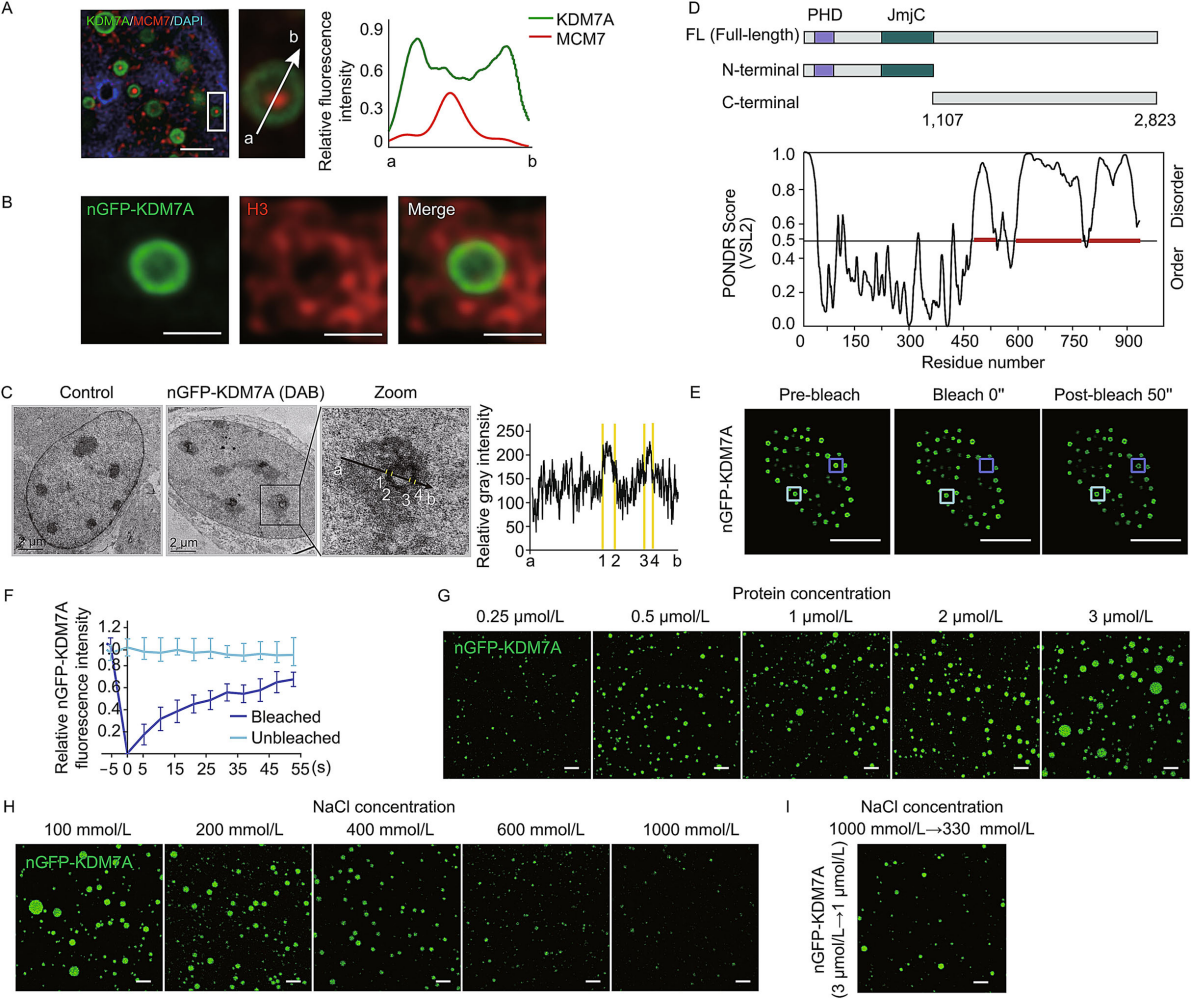

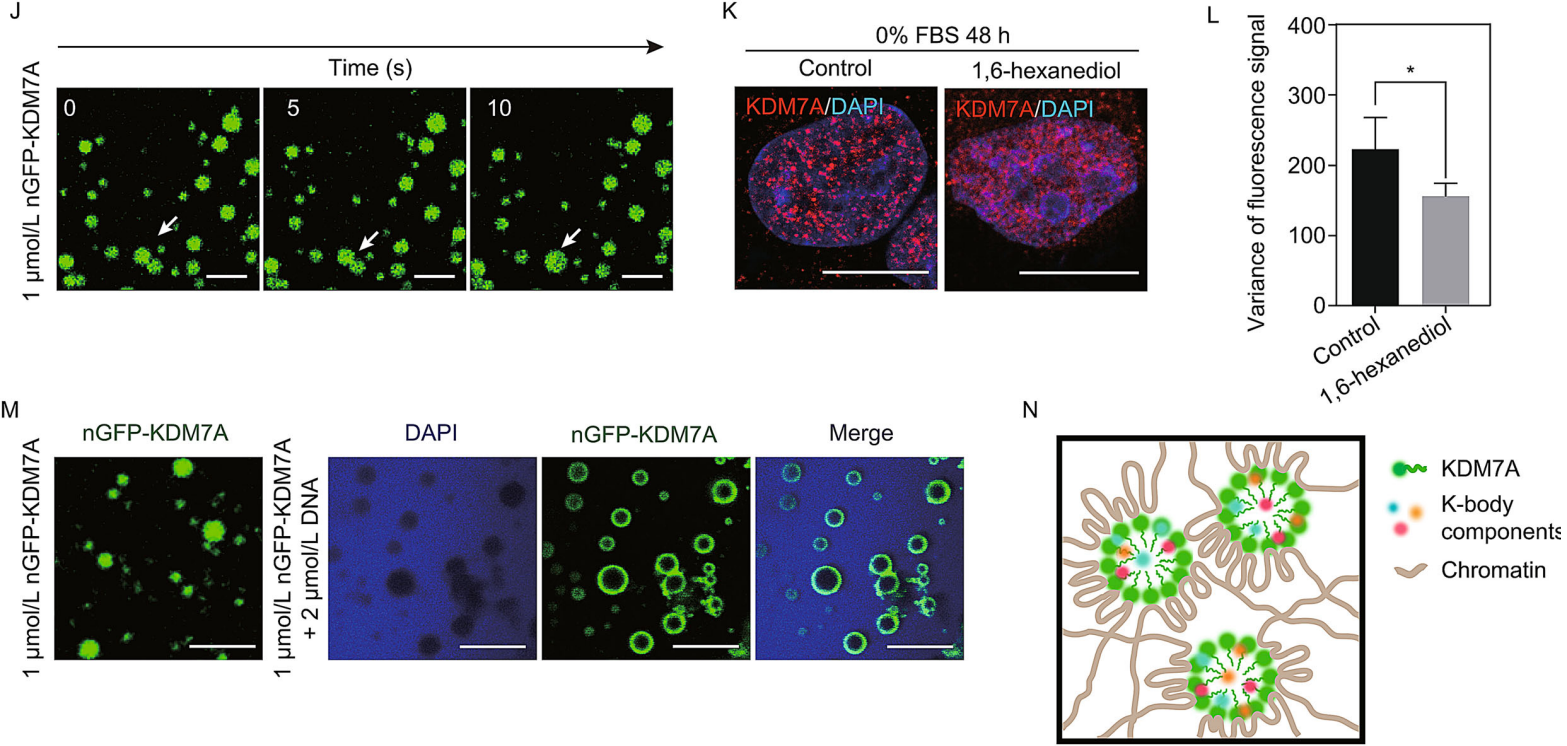
K-bodies are formed through liquid-liquid phase separation (LLPS). (A) Super-resolution fluorescence microscopy images showing localization of MCM7 relative to nGFP-KDM7A. Left, representative IF images; middle, local magnification of the place marked by a white box; right, relative fluorescence intensity of KDM7A and MCM7, from a to b. Scale bar, 1 µm. (B) Super-resolution microscopy images of immunofluorescence with histone H3 antibody in NIH3T3 cells transfected with nGFP-KDM7A. Scale bar, 1 µm. (C) Representative electronic microscopic pictures of KDM7A in control and nGFP-KDM7A transfected NIH3T3 cells labeled with DAB. Left, cells without DAB label; Middle, nGFP-KDM7A signal labeled by DAB and magnified image. Scale bar, 2 µm; Right, relative gray intensity from a to b in the zoom image. The number 1 to 4 represent the area of nGFP-KDM7A passed by the arrow. (D) Schematic diagram of KDM7A domains and order/disorder prediction by the PONDR algorithm. (E) Representative images of FRAP experiments using U2OS cells transfected with nGFP-KDM7A. Dark blue box shows the punctum undergoing bleaching, while the light blue box shows a control area. (F) Quantification of FRAP data for nGFP-KDM7A puncta. For both bleached area and unbleached control, fluorescence intensities are normalized to the pre-bleach intensity. Data are plotted as the mean ± s.d. (*n* = 7). (G) Droplet formation of purified nGFP-KDM7A at different protein concentrations in 75 mmol/L NaCl. Scale bar, 10 µm. (H) Droplet formation of purified nGFP-KDM7A (3 µmol/L) at different NaCl concentrations. Scale bar, 10 µm. (I) Droplet reformation after changing NaCl to 330 mmol/L from 1,000 mmol/L. Scale bar, 10 µm. (J) A fusion event of nGFP-KDM7A droplet *in vitro*. Scale bar, 10 µm. (K) The localization of KDM7A in HeLa cells treated with or without 10% 1,6-hexanediol in serum starvation condition. Scale bar, 10 µm. (L) The variance of fluorescence signal of KDM7A in control and 1,6-hexanediol treated HeLa cells. *P < 0.05 by unpaired *t*-test. (M) Purified nGFP-KDM7A with or without DNA. DNA is stained with DAPI. Scale bar, 10 µm. (N) A hypothetical model for the organization of K-bodies

Next, we examined the mechanisms underlying the formation of K-bodies. Based on live-cell imaging of nGFP-KDM7A expressed by an inducible system, KDM7A condensates were highly dynamic (Fig. S6A and Video S1). The C-terminal of KDM7A contains an intrinsically disordered region (IDR) (Fig. 2D), which is suggestive of the capability for phase separation (Wang et al., 2020). Indeed, overexpression of the C-terminal alone was sufficient to induce NBs. Compared to those induced by full-length nGFP-KDM7A, their sizes reduced, and their number increased (Fig. S6B and S6C). However, the overexpression of the N-terminal portion of KDM7A does not form granules, and these truncated proteins co-localize to the nucleolus (Fig. S6D). Furthermore, fluorescence recovery after photo-bleaching (FRAP) assay further showed that the KDM7A condensates can recover after photo-bleaching (Fig. 2E and 2F). Consistently, the complete fusion event of KDM7A condensates can be observed by live-cell imaging (Fig. S6E).

Purified KDM7A proteins can self-assemble into droplets *in vitro*. The size of the droplets increases with the higher KDM7A concentrations (Fig. 2G), and the structures are destroyed in high salt concentrations (Fig. 2H). When the salt solution was diluted to a lower concentration (330 mmol/L NaCl), droplet reformation occurred (Fig. 2I). Furthermore, the *in vitro* droplets can be recovered after photo-bleaching (Fig. S6F), and a few droplets had fused into one (Fig. 2J), similar to that observed in cells. The KDM7A C-terminal-truncated protein can also form liquid droplets *in vitro* (Fig. S6G). After treatment with 1,6-hexanediol, the signal of KDM7A in serum starved HeLa cells became more dispersed (Fig. 2K and 2L). Taken together, the behavior of the K-bodies *in vivo* and *in vitro* are consistent with general principles of LLPS (Wang et al., 2020), demonstrating that K-bodies are formed through phase separation.

After incubating the nGFP-KDM7A protein with purified genomic DNA, nGFP-KDM7A assembled into hollow spheres *in vitro*, similar to the intracellular KDM7A condensates (Fig. S6H). DAPI staining revealed that DNA is located outside the KDM7A hollow spheres *in vitro* (Fig. 2M). After adding DNA, the half recovery time of the condensates becomes shorter, and the recovered fluorescence intensity is slightly lower, as revealed by FRAP data (Fig. S6I). The theoretical isoelectric point (pI) of the N-terminal and C-terminal (IDR) of KDM7A is 5.84 and 8.98, respectively, based on the protein identification and analysis tools on the ExPASy Server (Wilkins et al., 1999). Compared with proteins that cannot form hollow spheres with DNA *in vitro*, such as cGAS, whose pI of N-terminal and C-terminal is 10.91 and 9.12 (Du and Chen, 2018), respectively, the pI of C-terminal and N-terminal of KDM7A are very different. Besides, it had been reported that the telomere DNA of *Xenopus laevis* can form *de novo* extrachromosomal circles (Cohen and Mechali, 2002). A similar mechanism may apply for KDM7A: DNA forms circles firstly, presumably through repeat sequences. In the PH 7.5 reaction condition, DNA and N-terminal of KDM7A have negative charges, and KDM7A IDR has positive charges. IDR of KDM7A can be bound to DNA by electrostatic interaction, while the N-terminal of KDM7A rejects DNA, and finally formed hollow sphere structures. Taken together, the *in situ* and *in vitro* data indicated that chromatin served as a platform to support K-body organization.

We therefore propose a model of how K-bodies are organized (Fig. 2N): a high concentration of KDM7A initiates phase separation through its IDR, recruits other proteins to participate in the regulation of cell physiological processes, and chromatin may function as the scaffold of K-bodies that contain various components.

Functional studies have shown that KDM7A plays important roles in the neural system. Overexpression of KDM7A promotes neural induction in early chick embryos and neural differentiation during mouse early embryonic development, whereas knocking down KDM7A impairs neural plate formation or blocks neural differentiation (Huang et al., 2010). Besides the brain, KDM7A expresses in all the other examined mouse tissues (Fig. S7A). KDM7A foci can be detected in some tissues such as brain, muscle, and testis (Fig. S7B), and the most obvious KDM7A foci were seen in round and elongating spermatids in the testis (Fig. S7C), which is consistent with the highest expression of KDM7A in the testis (Fig. S7A). The brain and muscle are tissues that consume a large amount of energy and may experience short-term nutritional deficiencies; spermatids are haploid germ cells that require precise DNA repairing. Of note, KDM7A foci found in the testis are large and concentrated around DAPI-dense regions, which may be associated with the high expression level of KDM7A and highly compact chromatin structure in haploid spermatids (Xia et al., 2020). Further studies are needed to reveal physiological functions of these nuclear structures.

There is increasing evidence indicating the non-genetic functions of chromatin such as serving as the platform for nuclear architecture. Microinjection of bacteriophage DNA into *Xenopus* eggs resulted in the spontaneous formation of a bilayer membrane around the injected genetic material (Forbes et al., 1983). In this study, we demonstrated that chromatin works as platforms for the organization of K-bodies, thus providing new evidence supporting the non-genetic functions of chromatin in establishing NBs. On the other hand, histone modifications regulate chromatin compartmentalization by driving phase separation (Wang et al., 2019). Therefore, KDM7A, as a histone demethylase with phase separation ability, may be involved in the process of chromatin compartmentalization, which awaits further investigations.

## Electronic supplementary material

Below is the link to the electronic supplementary material.

Supplementary material 1 (PDF 1308 kb)
