# Supplementary material for: The nuclear bodies formed by histone demethylase KDM7A
Source: Protein Cell. 2020 Sep 16;12(4):297–304. doi: 10.1007/s13238-020-00783-x (PMC8019015; doi:10.1007/s13238-020-00783-x)
Supplement: Supplementary file 1 — Supplementary material 1 (PDF 1308 kb) [file 13238_2020_783_MOESM1_ESM.pdf]

Materials and methods

Cell culture and transfection.

The human osteosarcoma cell line U2OS, human cervical cancer cell line HeLa, mouse embryo fibroblast cell line NIH3T3, and human embryonic kidney HEK293T cell line were obtained from ATCC. The cells were cultured in DMEM (ThermoFisher Scientific) supplemented with 10% FBS and NEAA according to the provided protocol. For serum starvation, cells were cultured with DMEM without serum for several time points. For amino acids starvation, cells were cultured with medium without amino acids for 48h. For high salt treatment, cells were cultured with 190mM NaCl for 48h. For 1,6-hexanediol treatment, cells were treated with 10% 1,6-hexanediol for 2min at room temperature. Plasmids were transfected into cells using polyethyleneimine (PEI) according to the operation instructions.

Plasmid construction.

ORFs were amplified by PCR from cDNA (derived from AML12 cells cells). The PCR products were digested with *BamH*I (*Asc*I) and *Mlu*I and cloned into the retroviral vector Lv-EF1a-GFP-MCS-IRES-puro. For RNAi experiments, shRNA plasmids were constructed by cloning the target sequences to the pLKO.1-puro vector: GAAATTCCAGACAATGTT AGA (shKDM7A-1), GTATAACTTCCACATTACAGT (shKDM7A-2) and ACTCGACAC TATAGTATCTCA (shControl).

FRAP.

The FRAP assay was performed in a live-cell chamber with confocal laser scanning microscopy. The bleaching duration was about 4 s in an area of one nuclear body. Images were captured at 1.3-s interval for 40 time points. The data were processed with LAS AF Lite.

Live-cell imaging.

Live-cell imaging experiments were performed on a GE DeltaVision inverted microscope. Images of U2OS cells were captured every 10 min for a total of 10 h. The data were processed with the Volocity software.

Western blotting.

Cells lysates were separated by SDS-PAGE and then transferred onto polyvinylidene fluoride (PVDF) membranes (Millipore). After washing and blocking, the membranes were incubated with primary antibodies as follows: anti-β-actin (66009-1-Ig, Proteintech), anti-GAPDH (9600, AGOMA), anti-H3 (AF0009, Beyotime), anti-MCM7 (sc-9966, Santa Cruz) and anti-KDM7A (raised against amino acids 417-735 in rabbit, ABclonal). The blots were detected with HRP-coupled secondary antibodies.

Immunofluorescence.

Immunofluorescence was performed as described elsewhere (Zhang et al., 2015). Besides those used for western blotting, other primary antibodies were: anti-SC35 (ab11826, Abcam), anti-PML (A1184, ABclonal; 05-718, Merck Millipore), anti-PSPC1 (SC-374387, Santa Cruz), anti-COIL (SC-55594, Santa Cruz), anti-Sam68 (sc-1238, Santa Cruz), anti-BMI1 (sc-390443, Santa Cruz) anti-C23 (10556-1-AP, Proteintech), anti-H3 (ab1791, Abcam) and MCM7 (sc-9966, Santa Cruz). The slides were assessed with a confocal laser scanning microscopy (Leica, SP5 and SP8).

Cell cycle assay.

The samples were prepared with the cell cycle and apoptosis analysis kit (Beyotime), and then analyzed using a FACSCalibur flow cytometer (BD).

TEM with HRP-DAB.

The NIH3T3 cells and mouse testis (Department of Laboratory Animal Science provided guidance on the study protocol) were fixed with 4% formaldehyde and incubated with the KDM7A antibody. The primary antibody was detected using an HRP secondary antibody. Then, DAB (P0203, Beyotime) was used for the HRP staining, which is EM-visible. After that, the samples were prepared for conventional TEM. The sections were stained and imaged at the EM facility of ION (Institute of Neuroscience, Shanghai, China).

Immunoprecipitation and mass spectrometry.

The U2OS cells were transfected with GFP-only or GFP-KDM7A plasmids for 24 h and then harvested. The nuclear extracts were prepared from 20 million cells and were incubated with 20 μL GFP-TRAP_M (gtm-20, Chromotek) in 50 mM Tris HCl (pH 7.5), 125 mM NaCl, 0.2% NP-40, 5% glycerol, 1.5 mM $MgCl_2$, and protease inhibitors. The LC-MS/MS experiments and data analysis were performed as described elsewhere (Zhang et al., 2015). The experiments were conducted using three biological replicates, and the proteins appeared three times in the GFP-KDM7A group but not more than once in the GFP-only group were considered as candidates. For IP/MS assay in HeLa cells under 0% FBS, cells were harvested after cultured in 0% FBS for 48h, with IgG as control. The proteins appeared more than two times in KDM7A group but not more than once in control group were considered as candidates.

Protein purification.

The KDM7A and C-terminal of KDM7A cDNA was cloned into the pCAG-6xHis-GFP -KDM7A plasmid and then transfected into HEK293T cells. The proteins were purified with Ni Aogarose 6 FF (AOGMA) with the AKTA system (GE Healthcare Life Sciences). The proteins were then concentrated in an Amicon Ultracel-50K spin concentrator and was exchanged to storage buffer [50 mM Tris-HCl(pH 7.5), 200 mM NaCl, 1 mM DTT, 10% glycerol]. The concentrated proteins were stored in -80°C freezer after flash freezing in liquid nitrogen.

Liquid droplet assay.

For the droplet assay, proteins were dropped on the living cell chamber at concentrations of 0.25, 0.5, 1, 2, 3 μM in the reaction buffer [50 mM Tris-HCl (pH 7.5), 100 mM NaCl, 1 mM DTT], and then imaged with a confocal laser scanning microscopy (Leica, SP5). For DNA contained droplet assays, genomic DNA was extracted from NIH3T3 cells and was sheered by sonication to lengths within the range of 300-700 bp. DNA was added to the protein solution at different concentrations and then imaged with a confocal laser scanning microscope (Leica, SP5).

Supplementary figure legends

Fig. S1: KDM7A marks a type of nuclear bodies (K-bodies) in serum starved Hela cells.

(A) The separated images of co-staining of KDM7A and SC35, PSPC1, PML, Coilin, BMI1 and Sam68 in Hela cells treated with 0% FBS for 48h, respectively, in Figure 1D. Scale bar, 10 μm.

(B) Co-stain of KDM7A and Pol II in Hela cells treated with 0% FBS for 48h. Scale bar, 10 μm. Right, Relative fluorescence intensity of KDM7A and Pol II from a to b.

(C) Representative images of KDM7A in Hela cells treated with amino acids starvation for 0h and 48h. Scale bar, 10 μm.

(D) The sizes of KDM7A foci in Hela cells under normal and amino acids starvation condition. 16-18 cells were analyzed. ns $p>0.05$ by unpaired t-test.

(E) Representative images of KDM7A in Hela cells treated with 190mM NaCl for 0h and 48h. Scale bar, 10 μm.

(F) The sizes of KDM7A foci in Hela cells treated with 190mM NaCl for 0h and 48h. 16-20 cells were analyzed. ****$p<0.0001$ by unpaired t-test.

Fig.S2: KDM7A condensates in U2OS cells transfected with nGFP-KDM7A.

(A) Western blot of H3K9me2 and H3K27me2 in Control and nGFP-KDM7A transfected 293T cells. H3 as the loading control.

(B) The localization of nGFP-KDM7A expressed in U2OS cells. Scale bar, 10 μm.

(C) The validation of KDM7A antibody. Representative images of immunofluorescence with KDM7A antibody in U2OS cells transfected with nGFP-KDM7A. Right: Relative fluorescence intensity of nGFP-KDM7A and KDM7A antibody from a to b. Scale bar, 10 μm.

(D) The separated images of U2OS cells transfected with nGFP-KDM7A and stained with SC35, PSPC1, PML, Coilin, BMI1 and Sam68 in Figure 1E. Scale bar, 10 μm.

Fig. S3: K-bodies interact with proteins associated with important biological processes.

(A) Silver staining of SDS-PAGE gel separating proteins immunoprecipitated with GFP-trap in U2OS cells. GFP-only as the negative control.

(B) Clustering analysis of nGFP-KDM7A-associated proteins with normalized number of peptides detected by LC-MS. Three biological replicates were conducted for nGFP-KDM7A (K1-K3) and GFP-only (G1-G3), respectively.

(C) Gene Ontology (GO) analysis of biological processes of KDM7A-associated proteins in U2OS cells. The enriched terms are ranked by $-\log_{10}$ (p values).

(D) Venn diagram of overlap proteins of KDM7A-associated proteins in Hela cells under 0% FBS and U2OS cells transfected with nGFP-KDM7A.

Fig. S4: KDM7A is partially required for the formation of MCM7 puncta.

(A) The localization of KDM7A and HNRNPU in U2OS cells transfected with nGFP-KDM7A. Scale bar, 10 μm. Right, relative fluorescence intensity of KDM7A and HNRNPU from a to b.

(B) Co-staining of KDM7A and MCM7 in shControl, shKDM7A-1 and shKDM7A-2 Hela cells treated with 0% FBS for 48h. Scale bar, 10 μm.

(C) The relative size of MCM7 foci in shControl, shKDM7A-1 and shKDM7A-2 Hela cells

treated with 0% FBS for 48h. 15-16 cells were analyzed. ****p<0.0001 by unpaired t-test.

(D) Western blot of KDM7A and MCM7 in shControl Hela cells under 10% FBS and shControl, shKDM7A-1, shKDM7A-2 Hela cells under 0% FBS, respectively. GAPDH as the loading control.

Fig. S5: KDM7A condensates are surrounded by chromatin.

(A) Representative results of immunofluorescence analysis with H3 antibody in KDM7A transfected (up) and control (down) cell. Scale bar, 10 μm. Right, relative fluorescence intensity of H3 and KDM7A crossed by the arrow on the left.

Fig. S6: K-bodies are formed by LLPS.

(A) Schematic representation and representative images of the appearance process of K-bodies in the inducible U2OS cell line. Scale bar, 10 μm.

(B) Representative images of U2OS cells transfected with FL or C-terminal nGFP-KDM7A, respectively. Scale bar, 10 μm.

(C) Quantification of the number (left) and size (right) of granules formed by FL or C-terminal nGFP-KDM7A. A total of 11 cells about 200 nuclear bodies were counted for each group. *p<0.05, ****p<0.0001 by unpaired t-test.

(D) The N-terminal of KDM7A aggregates into the nucleoli as detected by C23 immunofluorescence. Scale bar, 10 μm.

(E) A representative fusion event of K-bodies in the U2OS nucleus. Scale bar, 10 μm.

(F) FRAP experiments of nGFP-KDM7A in vitro droplets. Left: Representative images of FRAP. Pink box shows the punctum undergoing bleaching, while the blue box shows a control area. Scale bar, 10 μm. Right: Quantification of FRAP data for nGFP-KDM7A droplet. For both bleached area and unbleached control fluorescence intensities are normalized to the pre-bleach intensity. Data are plotted as the mean ± s.d. (n=6).

(G) Droplet formation of purified nGFP-C-terminal using different protein concentrations in 75 mM NaCl. Scale bar, 10 μm.

(H) Representative images of nGFP-KDM7A droplets by adding 0.25-2 μM DNA (300-700 bp in length). NaCl, 50 mM; nGFP-KDM7A protein, 0.5 μM. Scale bar, 10 μm.

(I) FRAP experiment of nGFP-KDM7A in vitro condensates incubated with DNA. Left: Representative images of FRAP. Pink box shows the hollow sphere undergoing bleaching, while the blue box shows a control area. Scale bar, 10 μm. Right: Quantification of FRAP data for nGFP-KDM7A condensates. For both bleached area and unbleached control fluorescence intensities are normalized to the pre-bleach intensity. Data are plotted as the mean ± s.d. (n=6).

Fig. S7: K-bodies exist in vivo.

(A) The expression of KDM7A in several mouse tissues detected by western blotting. β-actin as loading control.

(B) Immunofluorescence with KDM7A antibody in mouse brain, muscle, and testis tissues. Blue is DAPI and red is endogenous KDM7A. Scale bar, 10 μm.

(C) Representative IF images of KDM7A in 12-week-old mouse testis. Round sperms (RS) and elongating sperms (ES) are shown with white arrows. Scale bar, 10 μm.

Figure S1

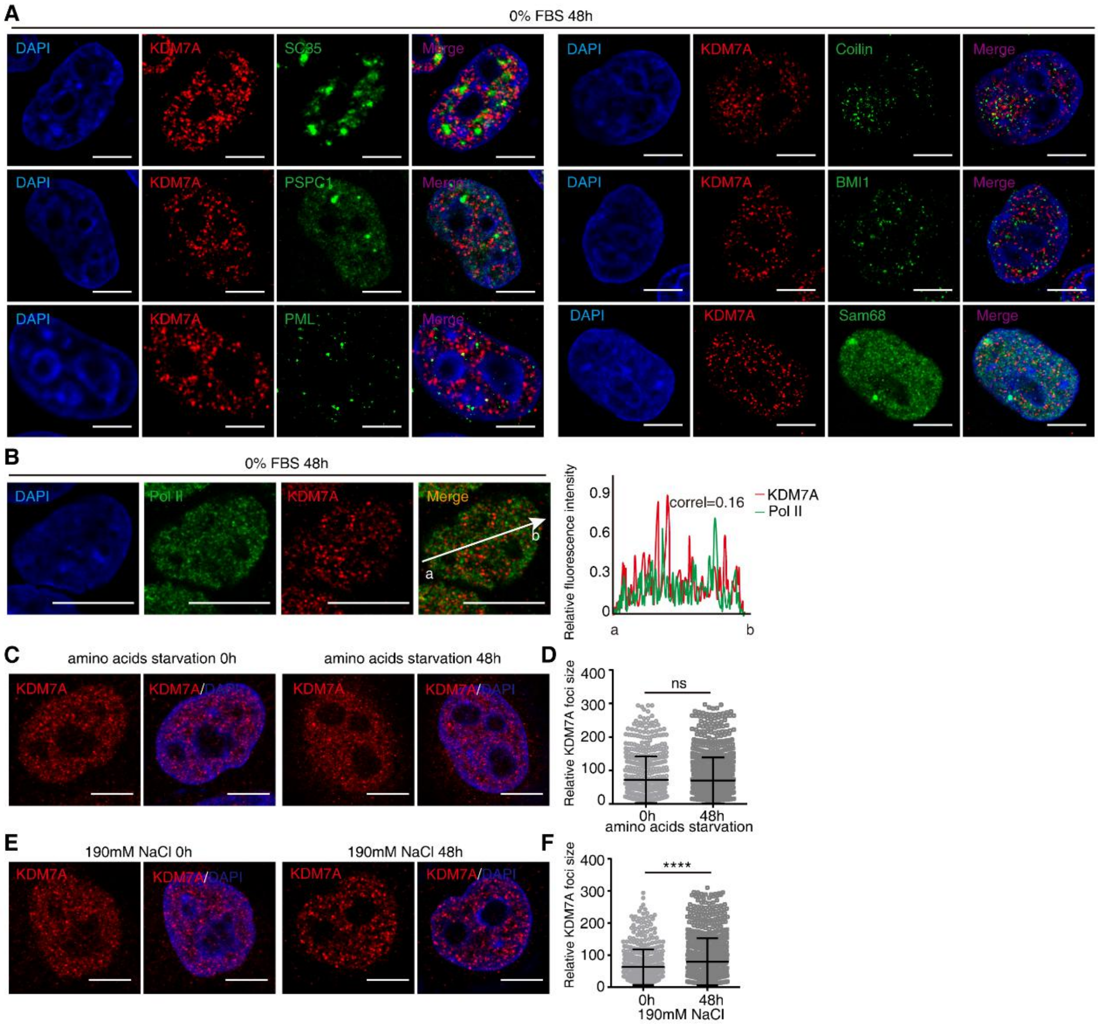

A
0% FBS 48h
DAPI
KDM7A
SC35
Merge
Coilin
PSPC1
BMI1
PML
Sam68
B
0% FBS 48h
Pol II
correl=0.16
Relative fluorescence intensity
0.9
0.6
0.3
0
a
b
C
amino acids starvation 0h
amino acids starvation 48h
KDM7A/DAPI
D
Relative KDM7A foci size
400
300
200
100
0
ns
0h
48h
amino acids starvation
E
190mM NaCl 0h
190mM NaCl 48h
F
****
190mM NaCl

Figure S2

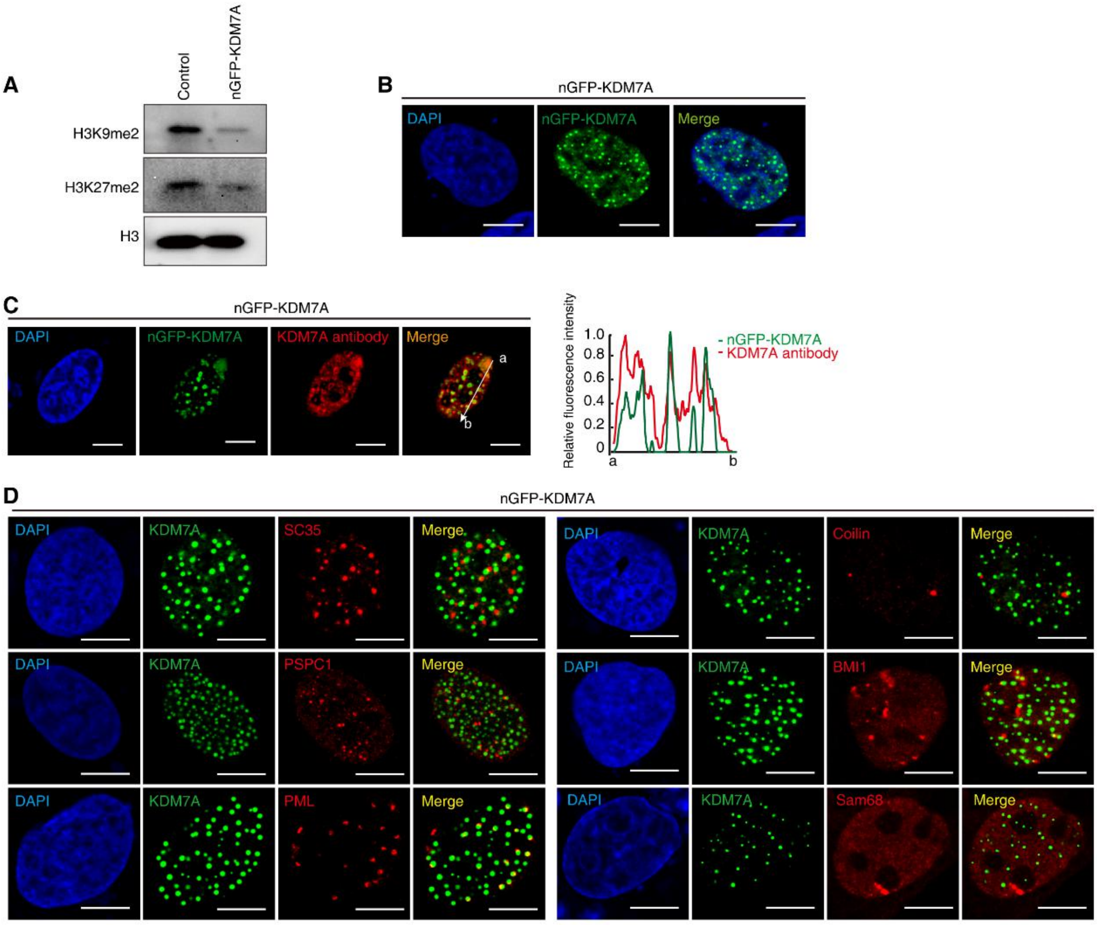
A
Control
nGFP-KDM7A
H3K9me2
H3K27me2
H3
B
nGFP-KDM7A
DAPI
nGFP-KDM7A
Merge
C
nGFP-KDM7A
DAPI
nGFP-KDM7A
KDM7A antibody
Merge
a
b
Relative fluorescence intensity
1.0
0.8
0.6
0.4
0.2
0
a
b
nGFP-KDM7A
KDM7A antibody
D
nGFP-KDM7A
DAPI
KDM7A
SC35
Merge
Coilin
PSPC1
BMI1
PML
Sam68

Figure S3

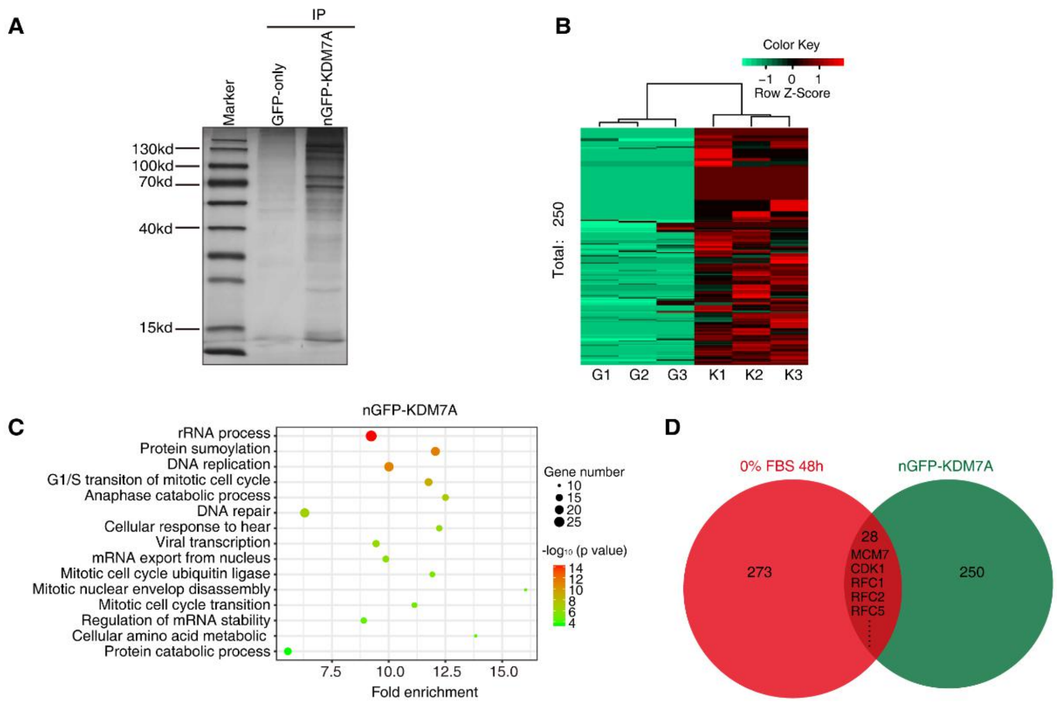
A
IP
Marker
GFP-only
nGFP-KDM7A
130kd
100kd
70kd
40kd
15kd
B
Color Key
−1 0 1
Row Z-Score
Total: 250
G1 G2 G3 K1 K2 K3
C
nGFP-KDM7A
rRNA process
Protein sumoylation
DNA replication
G1/S transiton of mitotic cell cycle
Anaphase catabolic process
DNA repair
Cellular response to hear
Viral transcription
mRNA export from nucleus
Mitotic cell cycle ubiquitin ligase
Mitotic nuclear envelop disassembly
Mitotic cell cycle transition
Regulation of mRNA stability
Cellular amino acid metabolic
Protein catabolic process
7.5 10.0 12.5 15.0
Fold enrichment
Gene number
10
15
20
25
-log10 (p value)
14
12
10
8
6
4
D
0% FBS 48h
nGFP-KDM7A
273
28
MCM7
CDK1
RFC1
RFC2
RFC5
250

Figure S4

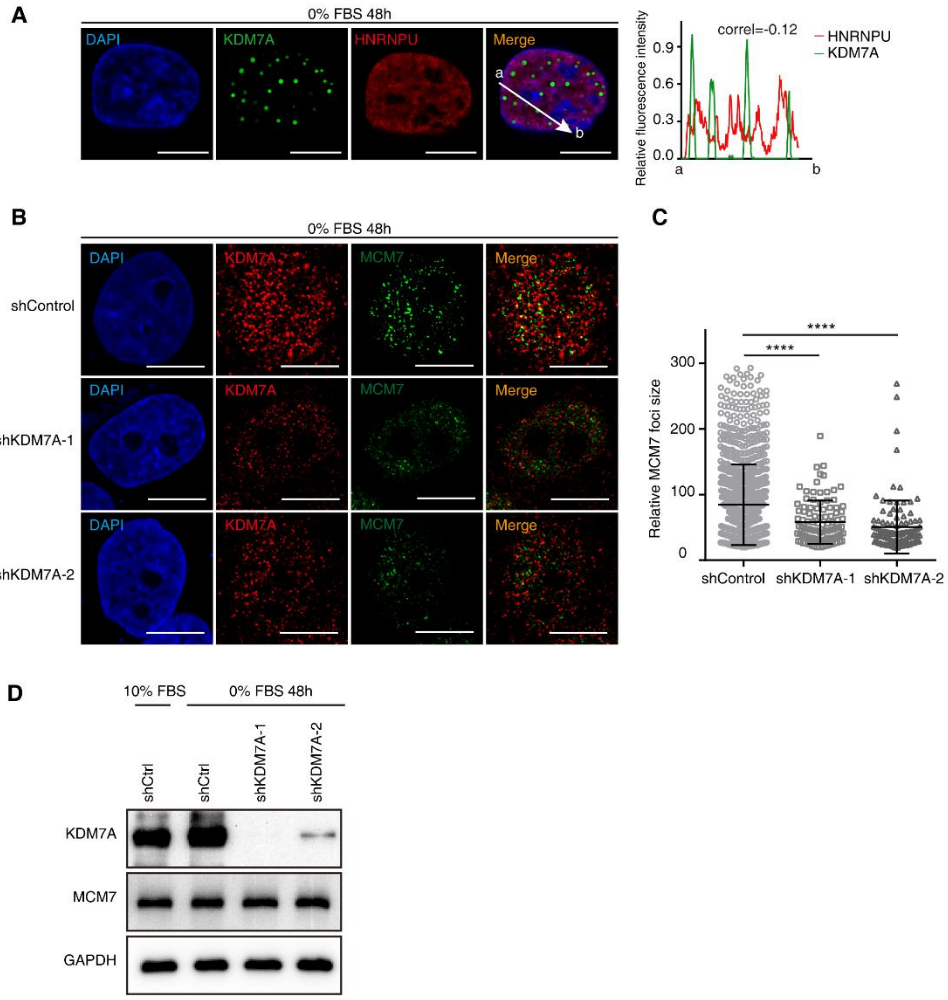
A
0% FBS 48h
DAPI
KDM7A
HNRNPU
Merge
a
b
correl=-0.12
HNRNPU
KDM7A
Relative fluorescence intensity
0.9
0.6
0.3
0.0
a
b
B
0% FBS 48h
shControl
shKDM7A-1
shKDM7A-2
DAPI
KDM7A
MCM7
Merge
C
****
****
Relative MCM7 foci size
300
200
100
0
shControl
shKDM7A-1
shKDM7A-2
D
10% FBS
0% FBS 48h
shCtrl
shCtrl
shKDM7A-1
shKDM7A-2
KDM7A
MCM7
GAPDH

Figure S5

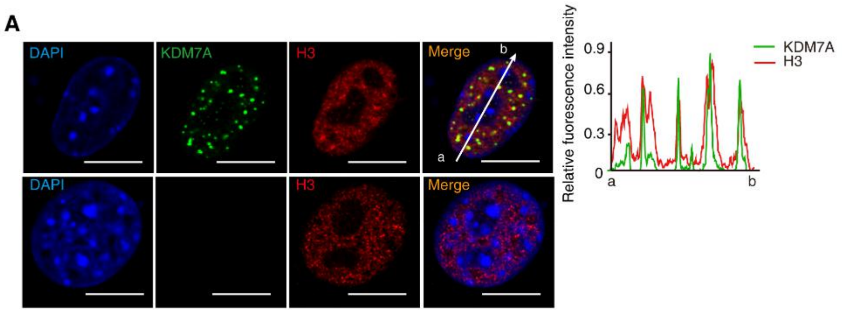
A
DAPI
KDM7A
H3
Merge
b
a
DAPI
H3
Merge
Relative fuorescence intensity
0.9
0.6
0.3
0
a
b
KDM7A
H3

Figure S6

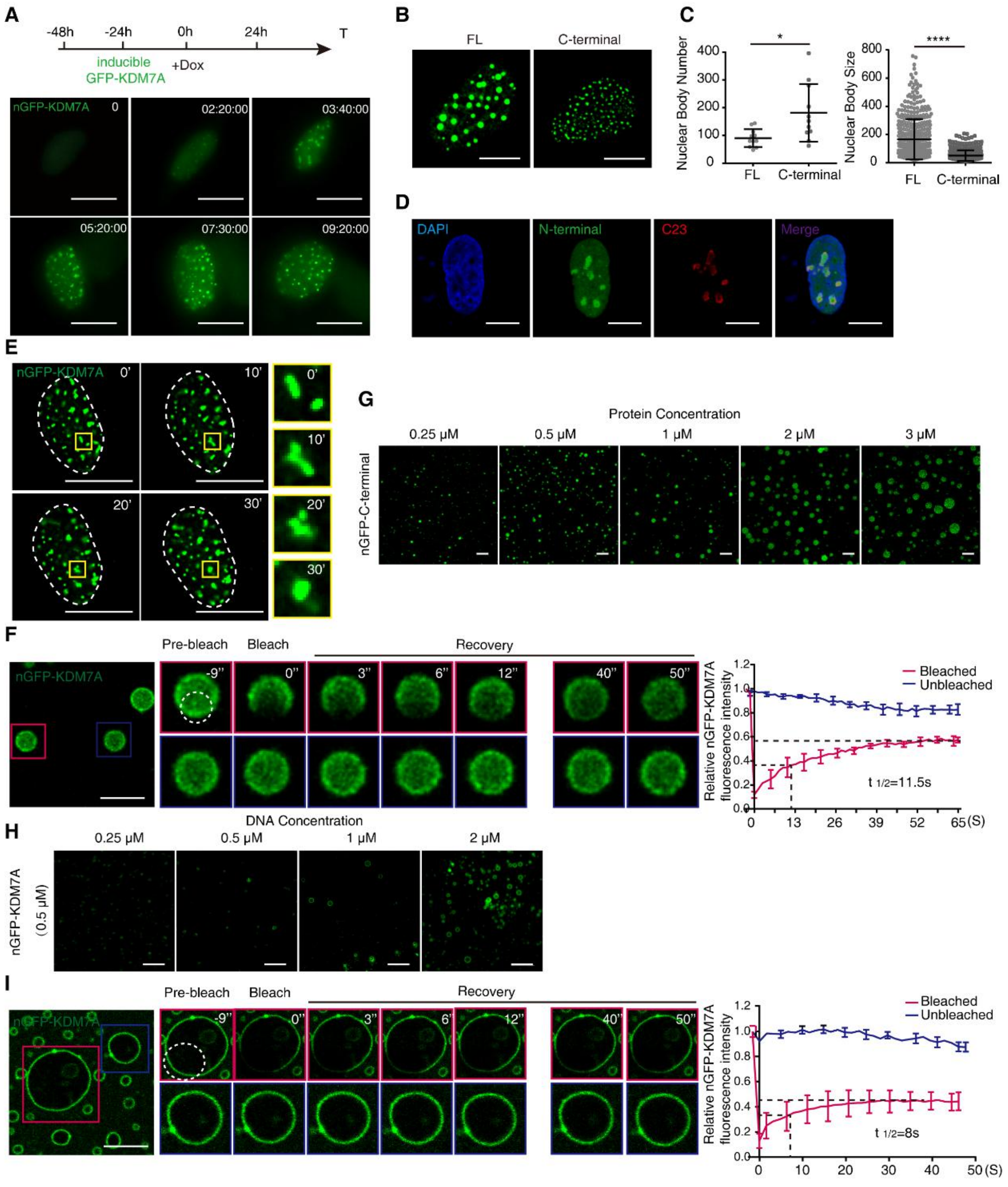
A
-48h
-24h
0h
24h
T
inducible
GFP-KDM7A
+Dox
nGFP-KDM7A
0
02:20:00
03:40:00
05:20:00
07:30:00
09:20:00
B
FL
C-terminal
C
Nuclear Body Number
FL
C-terminal
Nuclear Body Size
FL
C-terminal
D
DAPI
N-terminal
C23
Merge
E
nGFP-KDM7A
0'
10'
20'
30'
G
Protein Concentration
0.25 μM
0.5 μM
1 μM
2 μM
3 μM
nGFP-C-terminal
F
nGFP-KDM7A
Pre-bleach
Bleach
Recovery
-9''
0''
3''
6''
12''
40''
50''
Relative nGFP-KDM7A fluorescence intensity
Bleached
Unbleached
t 1/2=11.5s
(S)
H
DNA Concentration
0.25 μM
0.5 μM
1 μM
2 μM
nGFP-KDM7A
(0.5 μM)
I
nGFP-KDM7A
Pre-bleach
Bleach
Recovery
Relative nGFP-KDM7A fluorescence intensity
Bleached
Unbleached
t 1/2=8s
(S)

Figure S7

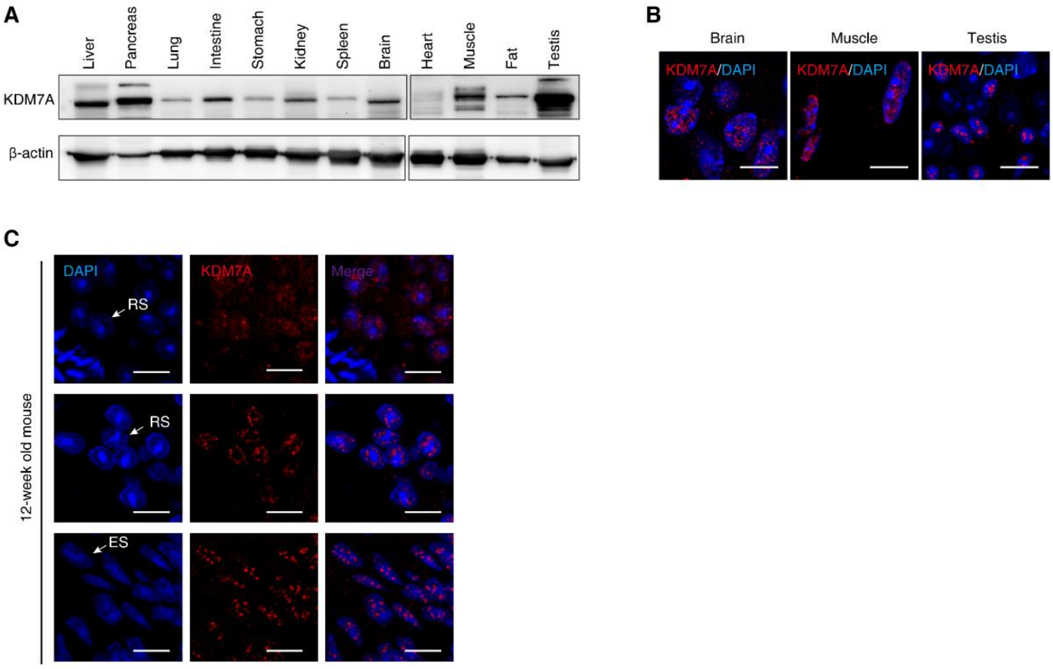
A
Liver
Pancreas
Lung
Intestine
Stomach
Kidney
Spleen
Brain
Heart
Muscle
Fat
Testis
KDM7A
β-actin
B
Brain
Muscle
Testis
KDM7A/DAPI
C
DAPI
KDM7A
Merge
RS
ES
12-week old mouse

Table S1. KDM7A associated proteins as identified by GFP-trap and mass spectrometry

Accession	Description	nGFP-KDM7A (Score)			GFP-Only (Score)		
		Rep 1	Rep 2	Rep 3	Rep 1	Rep 2	Rep 3
P33993	DNA replication licensing factor MCM7 OS=Homo sapiens GN=MCM7 PE=1 SV=4 - [MCM7_HUMAN]	628.7	721.8	719.1	0.0	0.0	0.0
Q96DF8	Protein DGCR14 OS=Homo sapiens GN=DGCR14 PE=1 SV=1 - [DGC14_HUMAN]	371.6	299.1	347.1	0.0	0.0	0.0
P28070	Proteasome subunit beta type-4 OS=Homo sapiens GN=PSMB4 PE=1 SV=4 - [PSB4_HUMAN]	323.9	243.4	266.6	0.0	0.0	0.0
Q96S55	ATPase WRNIP1 OS=Homo sapiens GN=WRNIP1 PE=1 SV=2 - [WRIP1_HUMAN]	268.4	312.5	253.8	0.0	0.0	0.0
P39748	Flap endonuclease 1 OS=Homo sapiens GN=FEN1 PE=1 SV=1 - [FEN1_HUMAN]	252.8	187.3	132.6	0.0	0.0	0.0
Q15773	Myeloid leukemia factor 2 OS=Homo sapiens GN=MLF2 PE=1 SV=1 - [MLF2_HUMAN]	250.2	174.1	135.8	0.0	0.0	0.0
Q7L590	Protein MCM10 homolog OS=Homo sapiens GN=MCM10 PE=1 SV=2 - [MCM10_HUMAN]	232.7	283.1	259.5	0.0	0.0	0.0
P04792	Heat shock protein beta-1 OS=Homo sapiens GN=HSPB1 PE=1 SV=2 - [HSPB1_HUMAN]	231.7	247.8	189.9	0.0	0.0	0.0
Q16576	Histone-binding protein RBBP7 OS=Homo sapiens GN=RBBP7 PE=1 SV=1 - [RBBP7_HUMAN]	230.4	211.9	186.9	0.0	0.0	0.0
P68371	Tubulin beta-4B chain OS=Homo sapiens GN=TUBB4B PE=1 SV=1 - [TBB4B_HUMAN]	229.5	290.2	197.0	0.0	0.0	0.0
Q04323	UBX domain-containing protein 1 OS=Homo sapiens GN=UBXN1 PE=1 SV=2 - [UBXN1_HUMAN]	221.1	128.3	151.5	0.0	0.0	0.0
P20618	Proteasome subunit beta type-1 OS=Homo sapiens GN=PSMB1 PE=1 SV=2 - [PSB1_HUMAN]	203.5	213.4	178.0	0.0	0.0	0.0
Q9Y2X9	Zinc finger protein 281 OS=Homo sapiens GN=ZNF281 PE=1 SV=1 - [ZN281_HUMAN]	196.3	131.1	162.4	0.0	0.0	0.0
P42771	Cyclin-dependent kinase inhibitor 2A, isoforms 1/2/3 OS=Homo sapiens GN=CDKN2A PE=1 SV=2 - [CD2A1_HUMAN]	196.1	105.3	38.0	0.0	0.0	0.0
Q6PD62	RNA polymerase-associated protein CTR9 homolog OS=Homo sapiens GN=CTR9 PE=1 SV=1 - [CTR9_HUMAN]	190.1	130.4	109.4	0.0	0.0	0.0
O75925	E3 SUMO-protein ligase PIAS1 OS=Homo sapiens GN=PIAS1 PE=1 SV=2 - [PIAS1_HUMAN]	187.8	139.2	38.2	0.0	0.0	0.0
O75190	DnaJ homolog subfamily B member 6 OS=Homo sapiens GN=DNAJB6 PE=1 SV=2 - [DNJB6_HUMAN]	187.6	202.0	138.7	0.0	0.0	0.0
O75419	Cell division control protein 45 homolog OS=Homo sapiens GN=CDC45 PE=1 SV=1 -	176.4	131.4	129.7	0.0	0.0	0.0

	[CDC45_HUMAN]						
P28066	Proteasome subunit alpha type-5 OS=Homo sapiens GN=PSMA5 PE=1 SV=3 - [PSA5_HUMAN]	167.5	106.8	184.2	0.0	0.0	0.0
P06493	Cyclin-dependent kinase 1 OS=Homo sapiens GN=CDK1 PE=1 SV=3 - [CDK1_HUMAN]	166.5	202.3	216.9	0.0	0.0	0.0
P55072	Transitional endoplasmic reticulum ATPase OS=Homo sapiens GN=VCP PE=1 SV=4 - [TERA_HUMAN]	163.4	114.2	45.0	0.0	0.0	0.0
P08238	Heat shock protein HSP 90-beta OS=Homo sapiens GN=HSP90AB1 PE=1 SV=4 - [HS90B_HUMAN]	156.5	123.3	88.1	0.0	0.0	0.0
O75694	Nuclear pore complex protein Nup155 OS=Homo sapiens GN=NUP155 PE=1 SV=1 - [NU155_HUMAN]	154.2	90.3	157.1	0.0	0.0	0.0
Q86Y56	HEAT repeat-containing protein 2 OS=Homo sapiens GN=HEATR2 PE=1 SV=4 - [HEAT2_HUMAN]	153.3	164.5	118.5	0.0	0.0	0.0
P49720	Proteasome subunit beta type-3 OS=Homo sapiens GN=PSMB3 PE=1 SV=2 - [PSB3_HUMAN]	148.8	102.4	187.1	0.0	0.0	0.0
O43242	26S proteasome non-ATPase regulatory subunit 3 OS=Homo sapiens GN=PSMD3 PE=1 SV=2 - [PSMD3_HUMAN]	145.5	91.1	76.5	0.0	0.0	0.0
O96000	NADH dehydrogenase [ubiquinone] 1 beta subcomplex subunit 10 OS=Homo sapiens GN=NDUFB10 PE=1 SV=3 - [NDUBA_HUMAN]	142.2	105.6	34.9	0.0	0.0	0.0
P61956	Small ubiquitin-related modifier 2 OS=Homo sapiens GN=SUMO2 PE=1 SV=3 - [SUMO2_HUMAN]	137.0	138.7	96.9	0.0	0.0	0.0
Q99956	Dual specificity protein phosphatase 9 OS=Homo sapiens GN=DUSP9 PE=1 SV=1 - [DUS9_HUMAN]	135.7	169.8	76.0	0.0	0.0	0.0
P49368	T-complex protein 1 subunit gamma OS=Homo sapiens GN=CCT3 PE=1 SV=4 - [TCPG_HUMAN]	127.4	94.2	85.5	0.0	0.0	0.0
P35251	Replication factor C subunit 1 OS=Homo sapiens GN=RFC1 PE=1 SV=4 - [RFC1_HUMAN]	127.4	39.8	65.7	0.0	0.0	0.0
Q9H0U9	Testis-specific Y-encoded-like protein 1 OS=Homo sapiens GN=TSPYL1 PE=1 SV=3 - [TSYL1_HUMAN]	127.3	159.4	122.4	0.0	0.0	0.0
P35453	Homeobox protein Hox-D13 OS=Homo sapiens GN=HOXD13 PE=1 SV=3 - [HXD13_HUMAN]	124.5	94.6	68.3	0.0	0.0	0.0
Q9Y4R8	Telomere length regulation protein TEL2 homolog OS=Homo sapiens GN=TELO2 PE=1 SV=2 - [TELO2_HUMAN]	123.3	139.1	142.5	0.0	0.0	0.0
Q6PJG6	BRCA1-associated ATM activator 1 OS=Homo	120.7	172.1	123.7	0.0	0.0	0.0

	sapiens GN=BRAT1 PE=1 SV=2 - [BRAT1_HUMAN]						
P08758	Annexin A5 OS=Homo sapiens GN=ANXA5 PE=1 SV=2 - [ANXA5_HUMAN]	119.0	108.9	88.8	0.0	0.0	0.0
Q8WV22	Non-structural maintenance of chromosomes element 1 homolog OS=Homo sapiens GN=NSMCE1 PE=1 SV=5 - [NSE1_HUMAN]	116.7	122.0	87.5	0.0	0.0	0.0
P49721	Proteasome subunit beta type-2 OS=Homo sapiens GN=PSMB2 PE=1 SV=1 - [PSB2_HUMAN]	114.2	145.7	106.2	0.0	0.0	0.0
Q6NSI4	Uncharacterized protein CXorf57 OS=Homo sapiens GN=CXorf57 PE=1 SV=2 - [CX057_HUMAN]	112.5	70.8	118.6	0.0	0.0	0.0
Q96GA3	Protein LTV1 homolog OS=Homo sapiens GN=LTV1 PE=1 SV=1 - [LTV1_HUMAN]	108.0	55.4	73.3	0.0	0.0	0.0
Q8WXF1	Paraspeckle component 1 OS=Homo sapiens GN=PSPC1 PE=1 SV=1 - [PSPC1_HUMAN]	107.8	92.2	27.3	0.0	0.0	0.0
Q9NRX1	RNA-binding protein PNO1 OS=Homo sapiens GN=PNO1 PE=1 SV=1 - [PNO1_HUMAN]	107.2	102.6	83.0	0.0	0.0	0.0
P17987	T-complex protein 1 subunit alpha OS=Homo sapiens GN=TCP1 PE=1 SV=1 - [TCPA_HUMAN]	105.6	210.6	166.7	0.0	0.0	0.0
O95816	BAG family molecular chaperone regulator 2 OS=Homo sapiens GN=BAG2 PE=1 SV=1 - [BAG2_HUMAN]	104.6	38.3	77.8	0.0	0.0	0.0
Q6P1K2	Polyamine-modulated factor 1 OS=Homo sapiens GN=PMF1 PE=1 SV=2 - [PMF1_HUMAN]	102.6	52.9	62.7	0.0	0.0	0.0
P63104	14-3-3 protein zeta/delta OS=Homo sapiens GN=YWHAZ PE=1 SV=1 - [1433Z_HUMAN]	102.4	73.6	88.8	0.0	0.0	0.0
Q9NRZ9	Lymphoid-specific helicase OS=Homo sapiens GN=HELLS PE=1 SV=1 - [HELLS_HUMAN]	101.0	41.5	60.4	0.0	0.0	0.0
Q02241	Kinesin-like protein KIF23 OS=Homo sapiens GN=KIF23 PE=1 SV=3 - [KIF23_HUMAN]	100.9	58.0	60.1	0.0	0.0	0.0
Q9Y314	Nitric oxide synthase-interacting protein OS=Homo sapiens GN=NOSIP PE=1 SV=1 - [NOSIP_HUMAN]	99.7	106.2	75.6	0.0	0.0	0.0
Q9NYZ3	G2 and S phase-expressed protein 1 OS=Homo sapiens GN=GTSE1 PE=1 SV=3 - [GTSE1_HUMAN]	99.0	74.1	142.5	0.0	0.0	0.0
Q9UMY1	Nucleolar protein 7 OS=Homo sapiens GN=NOL7 PE=1 SV=2 - [NOL7_HUMAN]	98.7	68.7	53.0	0.0	0.0	0.0
Q15019	Septin-2 OS=Homo sapiens GN=SEPT2 PE=1 SV=1 - [SEPT2_HUMAN]	98.5	55.0	138.4	0.0	0.0	0.0
P40937	Replication factor C subunit 5 OS=Homo sapiens GN=RFC5 PE=1 SV=1 - [RFC5_HUMAN]	98.1	118.5	42.2	0.0	0.0	0.0

Q14974	Importin subunit beta-1 OS=Homo sapiens GN=KPNB1 PE=1 SV=2 - [IMB1_HUMAN]	97.9	113.5	107.1	0.0	0.0	0.0
Q9Y371	Endophilin-B1 OS=Homo sapiens GN=SH3GLB1 PE=1 SV=1 - [SHLB1_HUMAN]	95.2	114.7	127.5	0.0	0.0	0.0
Q6W2J9	BCL-6 corepressor OS=Homo sapiens GN=BCOR PE=1 SV=1 - [BCOR_HUMAN]	95.2	44.3	89.4	0.0	0.0	0.0
Q01082	Spectrin beta chain, non-erythrocytic 1 OS=Homo sapiens GN=SPTBN1 PE=1 SV=2 - [SPTB2_HUMAN]	94.6	136.1	37.9	0.0	0.0	0.0
P55854	Small ubiquitin-related modifier 3 OS=Homo sapiens GN=SUMO3 PE=1 SV=2 - [SUMO3_HUMAN]	94.5	78.8	106.9	0.0	0.0	0.0
O60493	Sorting nexin-3 OS=Homo sapiens GN=SNX3 PE=1 SV=3 - [SNX3_HUMAN]	93.7	103.5	22.0	0.0	0.0	0.0
P12277	Creatine kinase B-type OS=Homo sapiens GN=CKB PE=1 SV=1 - [KCRB_HUMAN]	91.8	199.5	165.7	0.0	0.0	0.0
P62191	26S protease regulatory subunit 4 OS=Homo sapiens GN=PSMC1 PE=1 SV=1 - [PRS4_HUMAN]	91.7	91.0	40.7	0.0	0.0	0.0
P48444	Coatomer subunit delta OS=Homo sapiens GN=ARCN1 PE=1 SV=1 - [COPD_HUMAN]	90.8	82.0	61.6	0.0	0.0	0.0
O14965	Aurora kinase A OS=Homo sapiens GN=AURKA PE=1 SV=2 - [AURKA_HUMAN]	90.1	70.6	68.4	0.0	0.0	0.0
Q5SY16	Polynucleotide 5'-hydroxyl-kinase NOL9 OS=Homo sapiens GN=NOL9 PE=1 SV=1 - [NOL9_HUMAN]	90.0	41.8	53.4	0.0	0.0	0.0
Q9UPP1	Histone lysine demethylase PHF8 OS=Homo sapiens GN=PHF8 PE=1 SV=3 - [PHF8_HUMAN]	89.1	153.2	272.0	0.0	0.0	0.0
Q9BXK1	Krueppel-like factor 16 OS=Homo sapiens GN=KLF16 PE=1 SV=1 - [KLF16_HUMAN]	87.1	83.3	61.1	0.0	0.0	0.0
O00231	26S proteasome non-ATPase regulatory subunit 11 OS=Homo sapiens GN=PSMD11 PE=1 SV=3 - [PSD11_HUMAN]	87.1	99.2	70.9	0.0	0.0	0.0
Q9BSV6	tRNA-splicing endonuclease subunit Sen34 OS=Homo sapiens GN=TSEN34 PE=1 SV=1 - [SEN34_HUMAN]	86.3	66.7	69.9	0.0	0.0	0.0
P25789	Proteasome subunit alpha type-4 OS=Homo sapiens GN=PSMA4 PE=1 SV=1 - [PSA4_HUMAN]	85.6	86.4	80.0	0.0	0.0	0.0
Q86U38	Nucleolar protein 9 OS=Homo sapiens GN=NOP9 PE=1 SV=1 - [NOP9_HUMAN]	85.4	75.8	42.7	0.0	0.0	0.0
Q8IY67	Ribonucleoprotein PTB-binding 1 OS=Homo sapiens GN=RAVER1 PE=1 SV=1 - [RAVR1_HUMAN]	84.9	185.8	151.6	0.0	0.0	0.0
Q15475	Homeobox protein SIX1 OS=Homo sapiens GN=SIX1 PE=1 SV=1 - [SIX1_HUMAN]	83.3	122.9	69.2	0.0	0.0	0.0

O43175	D-3-phosphoglycerate dehydrogenase OS=Homo sapiens GN=PHGDH PE=1 SV=4 - [SERA_HUMAN]	82.4	37.3	41.4	0.0	0.0	0.0
Q99832	T-complex protein 1 subunit eta OS=Homo sapiens GN=CCT7 PE=1 SV=2 - [TCPH_HUMAN]	82.0	69.1	21.1	0.0	0.0	0.0
P19388	DNA-directed RNA polymerases I, II, and III subunit RPABC1 OS=Homo sapiens GN=POLR2E PE=1 SV=4 - [RPAB1_HUMAN]	79.9	94.8	60.1	0.0	0.0	0.0
P63165	Small ubiquitin-related modifier 1 OS=Homo sapiens GN=SUMO1 PE=1 SV=1 - [SUMO1_HUMAN]	79.3	79.6	41.7	0.0	0.0	0.0
Q9GZM3	DNA-directed RNA polymerase II subunit RPB11-b1 OS=Homo sapiens GN=POLR2J2 PE=1 SV=2 - [RPB1B_HUMAN]	78.8	69.3	67.1	0.0	0.0	0.0
P33991	DNA replication licensing factor MCM4 OS=Homo sapiens GN=MCM4 PE=1 SV=5 - [MCM4_HUMAN]	78.8	43.8	40.6	0.0	0.0	0.0
Q8N726	Cyclin-dependent kinase inhibitor 2A, isoform 4 OS=Homo sapiens GN=CDKN2A PE=1 SV=2 - [CD2A2_HUMAN]	77.7	126.9	149.8	0.0	0.0	0.0
Q5JTH9	RRP12-like protein OS=Homo sapiens GN=RRP12 PE=1 SV=2 - [RRP12_HUMAN]	77.3	94.1	18.0	0.0	0.0	0.0
Q9Y5Q9	General transcription factor 3C polypeptide 3 OS=Homo sapiens GN=GTF3C3 PE=1 SV=1 - [TF3C3_HUMAN]	76.4	70.4	63.7	0.0	0.0	0.0
P49643	DNA primase large subunit OS=Homo sapiens GN=PRIM2 PE=1 SV=2 - [PRI2_HUMAN]	76.1	39.0	84.1	0.0	0.0	0.0
P29590	Protein PML OS=Homo sapiens GN=PML PE=1 SV=3 - [PML_HUMAN]	76.1	75.6	62.5	0.0	0.0	0.0
Q9Y3I1	F-box only protein 7 OS=Homo sapiens GN=FBXO7 PE=1 SV=1 - [FBX7_HUMAN]	75.2	143.8	99.2	0.0	0.0	0.0
P11802	Cyclin-dependent kinase 4 OS=Homo sapiens GN=CDK4 PE=1 SV=2 - [CDK4_HUMAN]	74.5	66.4	42.9	0.0	0.0	0.0
Q15126	Phosphomevalonate kinase OS=Homo sapiens GN=PMVK PE=1 SV=3 - [PMVK_HUMAN]	72.3	68.3	48.0	0.0	0.0	0.0
Q9H992	E3 ubiquitin-protein ligase MARCH7 OS=Homo sapiens GN=MARCH7 PE=1 SV=1 - [MARH7_HUMAN]	72.1	79.1	55.5	0.0	0.0	0.0
P17482	Homeobox protein Hox-B9 OS=Homo sapiens GN=HOXB9 PE=1 SV=2 - [HXB9_HUMAN]	71.4	49.9	62.9	0.0	0.0	0.0
Q9HCK8	Chromodomain-helicase-DNA-binding protein 8 OS=Homo sapiens GN=CHD8 PE=1 SV=5 - [CHD8_HUMAN]	69.2	176.4	125.2	0.0	0.0	0.0
Q9P2E9	Ribosome-binding protein 1 OS=Homo sapiens GN=RRBP1 PE=1 SV=4 - [RRBP1_HUMAN]	68.7	59.8	76.6	0.0	0.0	0.0

P00374	Dihydrofolate reductase OS=Homo sapiens GN=DHFR PE=1 SV=2 - [DYR_HUMAN]	68.2	96.4	51.4	0.0	0.0	0.0
Q9P287	BRCA2 and CDKN1A-interacting protein OS=Homo sapiens GN=BCCIP PE=1 SV=1 - [BCCIP_HUMAN]	68.2	52.0	56.1	0.0	0.0	0.0
P35548	Homeobox protein MSX-2 OS=Homo sapiens GN=MSX2 PE=1 SV=3 - [MSX2_HUMAN]	68.1	65.6	67.9	0.0	0.0	0.0
Q5SSJ5	Heterochromatin protein 1-binding protein 3 OS=Homo sapiens GN=HP1BP3 PE=1 SV=1 - [HP1B3_HUMAN]	67.1	67.9	49.4	0.0	0.0	0.0
Q6P2C8	Mediator of RNA polymerase II transcription subunit 27 OS=Homo sapiens GN=MED27 PE=1 SV=1 - [MED27_HUMAN]	66.4	71.2	63.7	0.0	0.0	0.0
P46779	60S ribosomal protein L28 OS=Homo sapiens GN=RPL28 PE=1 SV=3 - [RL28_HUMAN]	66.0	68.6	44.5	0.0	0.0	0.0
P00492	Hypoxanthine-guanine phosphoribosyltransferase OS=Homo sapiens GN=HPRT1 PE=1 SV=2 - [HPRT_HUMAN]	65.4	47.9	104.8	0.0	0.0	0.0
Q96P11	Putative methyltransferase NSUN5 OS=Homo sapiens GN=NSUN5 PE=1 SV=2 - [NSUN5_HUMAN]	64.4	39.6	20.4	0.0	0.0	0.0
Q00013	55 kDa erythrocyte membrane protein OS=Homo sapiens GN=MPP1 PE=1 SV=2 - [EM55_HUMAN]	63.7	64.4	35.3	0.0	0.0	0.0
Q9Y285	Phenylalanine--tRNA ligase alpha subunit OS=Homo sapiens GN=FARSA PE=1 SV=3 - [SYFA_HUMAN]	63.5	103.9	84.5	0.0	0.0	0.0
Q8TBK6	Zinc finger CCHC domain-containing protein 10 OS=Homo sapiens GN=ZCCHC10 PE=2 SV=1 - [ZCH10_HUMAN]	63.1	72.5	49.8	0.0	0.0	0.0
Q96IY1	Kinetochore-associated protein NSL1 homolog OS=Homo sapiens GN=NSL1 PE=1 SV=3 - [NSL1_HUMAN]	62.4	79.8	54.5	0.0	0.0	0.0
Q8WUU5	GATA zinc finger domain-containing protein 1 OS=Homo sapiens GN=GATAD1 PE=1 SV=1 - [GATD1_HUMAN]	62.2	85.2	63.8	0.0	0.0	0.0
Q9ULX3	RNA-binding protein NOB1 OS=Homo sapiens GN=NOB1 PE=1 SV=1 - [NOB1_HUMAN]	61.7	80.7	32.2	0.0	0.0	0.0
P27694	Replication protein A 70 kDa DNA-binding subunit OS=Homo sapiens GN=RPA1 PE=1 SV=2 - [RFA1_HUMAN]	61.6	105.5	44.7	0.0	0.0	0.0
Q86X53	Glutamate-rich protein 1 OS=Homo sapiens GN=ERICH1 PE=1 SV=1 - [ERIC1_HUMAN]	60.1	65.1	24.1	0.0	0.0	0.0
P52701	DNA mismatch repair protein Msh6 OS=Homo sapiens GN=MSH6 PE=1 SV=2 - [MSH6_HUMAN]	59.8	30.3	41.8	0.0	0.0	0.0

Q92620	Pre-mRNA-splicing factor ATP-dependent RNA helicase PRP16 OS=Homo sapiens GN=DHX38 PE=1 SV=2 - [PRP16_HUMAN]	59.7	71.7	35.0	0.0	0.0	0.0
Q96J01	THO complex subunit 3 OS=Homo sapiens GN=THOC3 PE=1 SV=1 - [THOC3_HUMAN]	59.6	55.3	52.5	0.0	0.0	0.0
Q9BTT0	Acidic leucine-rich nuclear phosphoprotein 32 family member E OS=Homo sapiens GN=ANP32E PE=1 SV=1 - [AN32E_HUMAN]	59.6	81.1	78.1	0.0	0.0	0.0
P62899	60S ribosomal protein L31 OS=Homo sapiens GN=RPL31 PE=1 SV=1 - [RL31_HUMAN]	59.6	97.3	43.7	0.0	0.0	0.0
P12004	Proliferating cell nuclear antigen OS=Homo sapiens GN=PCNA PE=1 SV=1 - [PCNA_HUMAN]	59.1	44.1	62.6	0.0	0.0	0.0
Q99986	Serine/threonine-protein kinase VRK1 OS=Homo sapiens GN=VRK1 PE=1 SV=1 - [VRK1_HUMAN]	58.9	57.2	61.5	0.0	0.0	0.0
Q8NFH4	Nucleoporin Nup37 OS=Homo sapiens GN=NUP37 PE=1 SV=1 - [NUP37_HUMAN]	58.4	34.6	44.1	0.0	0.0	0.0
Q9UGL1	Lysine-specific demethylase 5B OS=Homo sapiens GN=KDM5B PE=1 SV=3 - [KDM5B_HUMAN]	58.0	96.5	75.9	0.0	0.0	0.0
O95071	E3 ubiquitin-protein ligase UBR5 OS=Homo sapiens GN=UBR5 PE=1 SV=2 - [UBR5_HUMAN]	57.8	77.2	32.6	0.0	0.0	0.0
Q8IUF8	Bifunctional lysine-specific demethylase and histidyl-hydroxylase MINA OS=Homo sapiens GN=MINA PE=1 SV=1 - [MINA_HUMAN]	55.9	81.6	77.5	0.0	0.0	0.0
P47914	60S ribosomal protein L29 OS=Homo sapiens GN=RPL29 PE=1 SV=2 - [RL29_HUMAN]	55.5	61.6	56.9	0.0	0.0	0.0
Q9BTE7	DCN1-like protein 5 OS=Homo sapiens GN=DCUN1D5 PE=1 SV=1 - [DCNL5_HUMAN]	55.0	82.1	73.8	0.0	0.0	0.0
O75792	Ribonuclease H2 subunit A OS=Homo sapiens GN=RNASEH2A PE=1 SV=2 - [RNH2A_HUMAN]	53.7	86.8	111.2	0.0	0.0	0.0
Q14166	Tubulin--tyrosine ligase-like protein 12 OS=Homo sapiens GN=TTLL12 PE=1 SV=2 - [TTL12_HUMAN]	53.3	60.5	24.8	0.0	0.0	0.0
Q99615	DnaJ homolog subfamily C member 7 OS=Homo sapiens GN=DNAJC7 PE=1 SV=2 - [DNJC7_HUMAN]	53.2	44.3	38.9	0.0	0.0	0.0
P46736	Lys-63-specific deubiquitinase BRCC36 OS=Homo sapiens GN=BRCC3 PE=1 SV=2 - [BRCC3_HUMAN]	52.8	103.9	75.0	0.0	0.0	0.0
P28340	DNA polymerase delta catalytic subunit OS=Homo sapiens GN=POLD1 PE=1 SV=2 - [DPOD1_HUMAN]	52.7	24.7	40.4	0.0	0.0	0.0
P49770	Translation initiation factor eIF-2B subunit beta OS=Homo sapiens GN=EIF2B2 PE=1 SV=3 - [EI2BB_HUMAN]	52.3	78.3	53.7	0.0	0.0	0.0

P33981	Dual specificity protein kinase TTK OS=Homo sapiens GN=TTK PE=1 SV=2 - [TTK_HUMAN]	51.7	61.7	33.5	0.0	0.0	0.0
Q9Y3A2	Probable U3 small nucleolar RNA-associated protein 11 OS=Homo sapiens GN=UTP11L PE=1 SV=2 - [UTP11_HUMAN]	51.7	54.4	62.1	0.0	0.0	0.0
Q9HAF1	Chromatin modification-related protein MEAF6 OS=Homo sapiens GN=MEAF6 PE=1 SV=1 - [EAF6_HUMAN]	51.3	71.0	68.3	0.0	0.0	0.0
Q9BSM1	Polycomb group RING finger protein 1 OS=Homo sapiens GN=PCGF1 PE=1 SV=2 - [PCGF1_HUMAN]	51.1	72.3	52.9	0.0	0.0	0.0
P12814	Alpha-actinin-1 OS=Homo sapiens GN=ACTN1 PE=1 SV=2 - [ACTN1_HUMAN]	51.0	43.6	71.6	0.0	0.0	0.0
P04818	Thymidylate synthase OS=Homo sapiens GN=TYMS PE=1 SV=3 - [TYSY_HUMAN]	50.9	50.3	88.6	0.0	0.0	0.0
Q96ST3	Paired amphipathic helix protein Sin3a OS=Homo sapiens GN=SIN3A PE=1 SV=2 - [SIN3A_HUMAN]	50.9	74.0	24.9	0.0	0.0	0.0
Q5W0B1	RING finger protein 219 OS=Homo sapiens GN=RNF219 PE=1 SV=1 - [RN219_HUMAN]	50.5	67.0	43.6	0.0	0.0	0.0
Q7Z7K6	Centromere protein V OS=Homo sapiens GN=CENPV PE=1 SV=1 - [CENPV_HUMAN]	49.8	139.9	78.0	0.0	0.0	0.0
Q6DKI1	60S ribosomal protein L7-like 1 OS=Homo sapiens GN=RPL7L1 PE=1 SV=1 - [RL7L_HUMAN]	49.2	50.9	41.4	0.0	0.0	0.0
P05386	60S acidic ribosomal protein P1 OS=Homo sapiens GN=RPLP1 PE=1 SV=1 - [RLA1_HUMAN]	48.7	48.8	48.7	0.0	0.0	0.0
Q99598	Translin-associated protein X OS=Homo sapiens GN=TSNAX PE=1 SV=1 - [TSNAX_HUMAN]	48.2	*	36.4	0.0	0.0	0.0
Q14781	Chromobox protein homolog 2 OS=Homo sapiens GN=CBX2 PE=1 SV=2 - [CBX2_HUMAN]	47.8	47.6	27.0	0.0	0.0	0.0
P60174	Triosephosphate isomerase OS=Homo sapiens GN=TPI1 PE=1 SV=3 - [TPIS_HUMAN]	47.4	48.5	45.8	0.0	0.0	0.0
P36543	V-type proton ATPase subunit E 1 OS=Homo sapiens GN=ATP6V1E1 PE=1 SV=1 - [VATE1_HUMAN]	47.4	38.1	76.1	0.0	0.0	0.0
Q9H147	Deoxynucleotidyltransferase terminal-interacting protein 1 OS=Homo sapiens GN=DNTTIP1 PE=1 SV=2 - [TDIF1_HUMAN]	47.0	30.5	85.2	0.0	0.0	0.0
P08243	Asparagine synthetase [glutamine-hydrolyzing] OS=Homo sapiens GN=ASNS PE=1 SV=4 - [ASNS_HUMAN]	46.4	39.6	76.6	0.0	0.0	0.0
Q9BQA1	Methylosome protein 50 OS=Homo sapiens GN=WDR77 PE=1 SV=1 - [MEP50_HUMAN]	46.3	46.2	46.5	0.0	0.0	0.0
P32969	60S ribosomal protein L9 OS=Homo sapiens GN=RPL9 PE=1 SV=1 - [RL9_HUMAN]	45.8	52.4	36.6	0.0	0.0	0.0

P36542	ATP synthase subunit gamma, mitochondrial OS=Homo sapiens GN=ATP5C1 PE=1 SV=1 - [ATPG_HUMAN]	45.6	56.2	30.6	0.0	0.0	0.0
P36639	7,8-dihydro-8-oxoguanine triphosphatase OS=Homo sapiens GN=NUDT1 PE=1 SV=3 - [8ODP_HUMAN]	45.4	49.8	45.6	0.0	0.0	0.0
P26641	Elongation factor 1-gamma OS=Homo sapiens GN=EEF1G PE=1 SV=3 - [EF1G_HUMAN]	45.2	63.4	60.4	0.0	0.0	0.0
P05198	Eukaryotic translation initiation factor 2 subunit 1 OS=Homo sapiens GN=EIF2S1 PE=1 SV=3 - [IF2A_HUMAN]	45.0	97.5	67.6	0.0	0.0	0.0
O43251	RNA binding protein fox-1 homolog 2 OS=Homo sapiens GN=RBFOX2 PE=1 SV=3 - [RFOX2_HUMAN]	44.0	95.9	65.3	0.0	0.0	0.0
O43913	Origin recognition complex subunit 5 OS=Homo sapiens GN=ORC5 PE=1 SV=1 - [ORC5_HUMAN]	43.6	70.6	59.2	0.0	0.0	0.0
Q13724	Mannosyl-oligosaccharide glucosidase OS=Homo sapiens GN=MOGS PE=1 SV=5 - [MOGS_HUMAN]	43.5	49.4	58.1	0.0	0.0	0.0
Q9H0E2	Toll-interacting protein OS=Homo sapiens GN=TOLLIP PE=1 SV=1 - [TOLIP_HUMAN]	42.6	42.8	47.3	0.0	0.0	0.0
P19623	Spermidine synthase OS=Homo sapiens GN=SRM PE=1 SV=1 - [SPEE_HUMAN]	42.4	36.8	37.2	0.0	0.0	0.0
Q5TA45	Integrator complex subunit 11 OS=Homo sapiens GN=CPSF3L PE=1 SV=2 - [INT11_HUMAN]	42.4	40.6	45.6	0.0	0.0	0.0
Q14738	Serine/threonine-protein phosphatase 2A 56 kDa regulatory subunit delta isoform OS=Homo sapiens GN=PPP2R5D PE=1 SV=1 - [2A5D_HUMAN]	41.9	37.8	32.7	0.0	0.0	0.0
Q9Y4C2	Protein FAM115A OS=Homo sapiens GN=FAM115A PE=1 SV=3 - [F115A_HUMAN]	41.3	52.6	57.7	0.0	0.0	0.0
Q5SRE5	Nucleoporin NUP188 homolog OS=Homo sapiens GN=NUP188 PE=1 SV=1 - [NU188_HUMAN]	41.1	47.4	47.2	0.0	0.0	0.0
Q8NCN4	E3 ubiquitin-protein ligase RNF169 OS=Homo sapiens GN=RNF169 PE=1 SV=2 - [RN169_HUMAN]	40.6	55.7	39.9	0.0	0.0	0.0
Q92979	Ribosomal RNA small subunit methyltransferase NEP1 OS=Homo sapiens GN=EMG1 PE=1 SV=4 - [NEP1_HUMAN]	40.3	62.9	55.2	0.0	0.0	0.0
Q86U06	Probable RNA-binding protein 23 OS=Homo sapiens GN=RBM23 PE=1 SV=1 - [RBM23_HUMAN]	40.2	77.2	64.6	0.0	0.0	0.0
Q9Y617	Phosphoserine aminotransferase OS=Homo sapiens GN=PSAT1 PE=1 SV=2 - [SERC_HUMAN]	39.4	46.6	32.1	0.0	0.0	0.0

Q14692	Ribosome biogenesis protein BMS1 homolog OS=Homo sapiens GN=BMS1 PE=1 SV=1 - [BMS1_HUMAN]	39.2	80.8	50.7	0.0	0.0	0.0
Q13309	S-phase kinase-associated protein 2 OS=Homo sapiens GN=SKP2 PE=1 SV=2 - [SKP2_HUMAN]	39.1	44.5	45.9	0.0	0.0	0.0
Q9BQ52	Zinc phosphodiesterase ELAC protein 2 OS=Homo sapiens GN=ELAC2 PE=1 SV=2 - [RNZ2_HUMAN]	38.6	26.9	53.5	0.0	0.0	0.0
Q12769	Nuclear pore complex protein Nup160 OS=Homo sapiens GN=NUP160 PE=1 SV=3 - [NU160_HUMAN]	38.3	26.0	45.0	0.0	0.0	0.0
P61160	Actin-related protein 2 OS=Homo sapiens GN=ACTR2 PE=1 SV=1 - [ARP2_HUMAN]	38.1	52.5	53.2	0.0	0.0	0.0
Q8WTT2	Nucleolar complex protein 3 homolog OS=Homo sapiens GN=NOC3L PE=1 SV=1 - [NOC3L_HUMAN]	37.0	52.5	52.6	0.0	0.0	0.0
Q13112	Chromatin assembly factor 1 subunit B OS=Homo sapiens GN=CHAF1B PE=1 SV=1 - [CAF1B_HUMAN]	33.9	51.7	58.1	0.0	0.0	0.0
P57678	Gem-associated protein 4 OS=Homo sapiens GN=GEMIN4 PE=1 SV=2 - [GEMI4_HUMAN]	33.4	70.8	40.1	0.0	0.0	0.0
O95478	Ribosome biogenesis protein NSA2 homolog OS=Homo sapiens GN=NSA2 PE=1 SV=1 - [NSA2_HUMAN]	33.1	62.7	61.8	0.0	0.0	0.0
Q99959	Plakophilin-2 OS=Homo sapiens GN=PKP2 PE=1 SV=2 - [PKP2_HUMAN]	32.7	61.4	40.5	0.0	0.0	0.0
P17980	26S protease regulatory subunit 6A OS=Homo sapiens GN=PSMC3 PE=1 SV=3 - [PRS6A_HUMAN]	32.4	23.4	60.9	0.0	0.0	0.0
P82930	28S ribosomal protein S34, mitochondrial OS=Homo sapiens GN=MRPS34 PE=1 SV=2 - [RT34_HUMAN]	31.8	42.8	38.9	0.0	0.0	0.0
Q14669	E3 ubiquitin-protein ligase TRIP12 OS=Homo sapiens GN=TRIP12 PE=1 SV=1 - [TRIPC_HUMAN]	31.8	73.1	74.4	0.0	0.0	0.0
Q96HA7	Tonsoku-like protein OS=Homo sapiens GN=TONSL PE=1 SV=2 - [TONSL_HUMAN]	30.9	25.7	82.8	0.0	0.0	0.0
Q6NXE6	Armadillo repeat-containing protein 6 OS=Homo sapiens GN=ARMC6 PE=1 SV=2 - [ARMC6_HUMAN]	30.9	78.0	123.1	0.0	0.0	0.0
Q9UKF6	Cleavage and polyadenylation specificity factor subunit 3 OS=Homo sapiens GN=CPSF3 PE=1 SV=1 - [CPSF3_HUMAN]	30.3	25.6	46.3	0.0	0.0	0.0
Q9BWN1	Proline-rich protein 14 OS=Homo sapiens GN=PRR14 PE=1 SV=1 - [PRR14_HUMAN]	29.5	52.0	38.5	0.0	0.0	0.0

Q12789	General transcription factor 3C polypeptide 1 OS=Homo sapiens GN=GTF3C1 PE=1 SV=4 - [TF3C1_HUMAN]	28.6	79.8	38.7	0.0	0.0	0.0
P11474	Steroid hormone receptor ERR1 OS=Homo sapiens GN=ESRRA PE=1 SV=3 - [ERR1_HUMAN]	28.0	24.8	43.1	0.0	0.0	0.0
O94761	ATP-dependent DNA helicase Q4 OS=Homo sapiens GN=RECQL4 PE=1 SV=1 - [RECQ4_HUMAN]	27.7	32.9	28.0	0.0	0.0	0.0
Q9H0W9	Ester hydrolase C11orf54 OS=Homo sapiens GN=C11orf54 PE=1 SV=1 - [CK054_HUMAN]	27.0	23.9	21.0	0.0	0.0	0.0
O15145	Actin-related protein 2/3 complex subunit 3 OS=Homo sapiens GN=ARPC3 PE=1 SV=3 - [ARPC3_HUMAN]	26.5	88.4	24.6	0.0	0.0	0.0
O95707	Ribonuclease P protein subunit p29 OS=Homo sapiens GN=POP4 PE=1 SV=2 - [RPP29_HUMAN]	25.3	25.6	27.8	0.0	0.0	0.0
Q99575	Ribonucleases P/MRP protein subunit POP1 OS=Homo sapiens GN=POP1 PE=1 SV=2 - [POP1_HUMAN]	24.3	30.0	66.2	0.0	0.0	0.0
Q53H80	Akirin-2 OS=Homo sapiens GN=AKIRIN2 PE=1 SV=2 - [AKIR2_HUMAN]	21.7	33.3	27.1	0.0	0.0	0.0
Q9H3M7	Thioredoxin-interacting protein OS=Homo sapiens GN=TXNIP PE=1 SV=1 - [TXNIP_HUMAN]	21.7	60.5	54.5	0.0	0.0	0.0
Q96T88	E3 ubiquitin-protein ligase UHRF1 OS=Homo sapiens GN=UHRF1 PE=1 SV=1 - [UHRF1_HUMAN]	181.3	192.4	146.1	137.4	0.0	0.0
Q9BQG0	Myb-binding protein 1A OS=Homo sapiens GN=MYBBP1A PE=1 SV=2 - [MBB1A_HUMAN]	148.0	348.2	246.4	78.8	0.0	0.0
Q9NXF1	Testis-expressed sequence 10 protein OS=Homo sapiens GN=TEX10 PE=1 SV=2 - [TEX10_HUMAN]	102.4	100.5	57.9	67.3	0.0	0.0
P47755	F-actin-capping protein subunit alpha-2 OS=Homo sapiens GN=CAPZA2 PE=1 SV=3 - [CAZA2_HUMAN]	68.8	53.1	58.8	62.4	0.0	0.0
Q9NRL2	Bromodomain adjacent to zinc finger domain protein 1A OS=Homo sapiens GN=BAZ1A PE=1 SV=2 - [BAZ1A_HUMAN]	54.7	139.0	107.4	59.5	0.0	0.0
Q5RKV6	Exosome complex component MTR3 OS=Homo sapiens GN=EXOSC6 PE=1 SV=1 - [EXOS6_HUMAN]	39.5	43.1	50.6	53.7	0.0	0.0
P56182	Ribosomal RNA processing protein 1 homolog A OS=Homo sapiens GN=RRP1 PE=1 SV=1 - [RRP1_HUMAN]	71.7	65.6	147.7	46.2	0.0	0.0
Q96EK4	THAP domain-containing protein 11 OS=Homo sapiens GN=THAP11 PE=1 SV=2 - [THA11_HUMAN]	66.5	70.4	57.2	40.9	0.0	0.0

Q13257	Mitotic spindle assembly checkpoint protein MAD2A OS=Homo sapiens GN=MAD2L1 PE=1 SV=1 - [MD2L1_HUMAN]	32.2	96.0	68.0	32.5	0.0	0.0
Q8IZL8	Proline-, glutamic acid- and leucine-rich protein 1 OS=Homo sapiens GN=PELP1 PE=1 SV=2 - [PELP1_HUMAN]	98.3	140.9	61.5	29.3	0.0	0.0
Q9Y4C8	Probable RNA-binding protein 19 OS=Homo sapiens GN=RBM19 PE=1 SV=3 - [RBM19_HUMAN]	33.4	81.3	98.5	26.6	0.0	0.0
Q92621	Nuclear pore complex protein Nup205 OS=Homo sapiens GN=NUP205 PE=1 SV=3 - [NU205_HUMAN]	58.0	61.2	40.9	25.7	0.0	0.0
O14744	Protein arginine N-methyltransferase 5 OS=Homo sapiens GN=PRMT5 PE=1 SV=4 - [ANM5_HUMAN]	21.5	75.7	24.7	0.0	0.0	0.0
P55795	Heterogeneous nuclear ribonucleoprotein H2 OS=Homo sapiens GN=HNRNPH2 PE=1 SV=1 - [HNRH2_HUMAN]	426.3	384.7	486.9	0.0	213.8	0.0
Q08170	Serine/arginine-rich splicing factor 4 OS=Homo sapiens GN=SRSF4 PE=1 SV=2 - [SRSF4_HUMAN]	78.6	75.9	122.5	0.0	179.5	0.0
Q13148	TAR DNA-binding protein 43 OS=Homo sapiens GN=TARDBP PE=1 SV=1 - [TADBP_HUMAN]	90.2	190.4	189.2	0.0	104.5	0.0
Q9Y262	Eukaryotic translation initiation factor 3 subunit L OS=Homo sapiens GN=EIF3L PE=1 SV=1 - [EIF3L_HUMAN]	117.0	110.5	134.7	0.0	101.9	0.0
O00303	Eukaryotic translation initiation factor 3 subunit F OS=Homo sapiens GN=EIF3F PE=1 SV=1 - [EIF3F_HUMAN]	39.3	88.6	105.9	0.0	81.0	0.0
Q9BYG3	MKI67 FHA domain-interacting nucleolar phosphoprotein OS=Homo sapiens GN=NIFK PE=1 SV=1 - [MK67I_HUMAN]	182.4	216.6	195.6	0.0	79.4	0.0
Q6RFH5	WD repeat-containing protein 74 OS=Homo sapiens GN=WDR74 PE=1 SV=1 - [WDR74_HUMAN]	109.4	98.7	81.2	0.0	66.3	0.0
Q7L0Y3	Mitochondrial ribonuclease P protein 1 OS=Homo sapiens GN=TRMT10C PE=1 SV=2 - [MRRP1_HUMAN]	111.6	142.1	68.1	0.0	59.2	0.0
P98175	RNA-binding protein 10 OS=Homo sapiens GN=RBM10 PE=1 SV=3 - [RBM10_HUMAN]	53.4	93.6	119.7	0.0	58.9	0.0
Q5C9Z4	Nucleolar MIF4G domain-containing protein 1 OS=Homo sapiens GN=NOM1 PE=1 SV=1 - [NOM1_HUMAN]	55.0	38.7	47.1	0.0	53.7	0.0
Q9BQ67	Glutamate-rich WD repeat-containing protein 1 OS=Homo sapiens GN=GRWD1 PE=1 SV=1 -	85.0	49.1	65.2	0.0	50.4	0.0

	[GRWD1_HUMAN]						
Q00059	Transcription factor A, mitochondrial OS=Homo sapiens GN=TFAM PE=1 SV=1 - [TFAM_HUMAN]	48.2	23.6	39.5	0.0	49.7	0.0
P35250	Replication factor C subunit 2 OS=Homo sapiens GN=RFC2 PE=1 SV=3 - [RFC2_HUMAN]	21.2	69.4	44.0	0.0	49.0	0.0
Q5UIP0	Telomere-associated protein RIF1 OS=Homo sapiens GN=RIF1 PE=1 SV=2 - [RIF1_HUMAN]	32.1	38.6	74.6	0.0	48.8	0.0
Q9NV31	U3 small nucleolar ribonucleoprotein protein IMP3 OS=Homo sapiens GN=IMP3 PE=1 SV=1 - [IMP3_HUMAN]	65.8	30.1	117.6	0.0	47.5	0.0
Q9Y6V7	Probable ATP-dependent RNA helicase DDX49 OS=Homo sapiens GN=DDX49 PE=1 SV=1 - [DDX49_HUMAN]	85.4	73.0	50.1	0.0	44.7	0.0
P46783	40S ribosomal protein S10 OS=Homo sapiens GN=RPS10 PE=1 SV=1 - [RS10_HUMAN]	134.3	212.3	230.3	0.0	43.4	0.0
O15381	Nuclear valosin-containing protein-like OS=Homo sapiens GN=NVL PE=1 SV=1 - [NVL_HUMAN]	181.7	239.9	251.3	0.0	39.5	0.0
Q8IWA0	WD repeat-containing protein 75 OS=Homo sapiens GN=WDR75 PE=1 SV=1 - [WDR75_HUMAN]	125.7	189.0	191.3	0.0	38.0	0.0
Q86W42	THO complex subunit 6 homolog OS=Homo sapiens GN=THOC6 PE=1 SV=1 - [THOC6_HUMAN]	91.6	136.5	142.0	0.0	37.5	0.0
Q7Z3B4	Nucleoporin p54 OS=Homo sapiens GN=NUP54 PE=1 SV=2 - [NUP54_HUMAN]	23.9	34.8	31.3	0.0	37.5	0.0
P42285	Superkiller viralicidic activity 2-like 2 OS=Homo sapiens GN=SKIV2L2 PE=1 SV=3 - [SK2L2_HUMAN]	89.7	60.6	61.0	0.0	36.8	0.0
Q969G3	SWI/SNF-related matrix-associated actin-dependent regulator of chromatin subfamily E member 1 OS=Homo sapiens GN=SMARCE1 PE=1 SV=2 - [SMCE1_HUMAN]	90.7	50.7	60.0	0.0	36.7	0.0
O00566	U3 small nucleolar ribonucleoprotein protein MPP10 OS=Homo sapiens GN=MPHOSPH10 PE=1 SV=2 - [MPP10_HUMAN]	49.7	112.9	89.9	0.0	34.8	0.0
Q96P16	Regulation of nuclear pre-mRNA domain-containing protein 1A OS=Homo sapiens GN=RPRD1A PE=1 SV=1 - [RPR1A_HUMAN]	123.5	77.5	48.0	0.0	34.7	0.0
Q96BK5	PIN2/TERF1-interacting telomerase inhibitor 1 OS=Homo sapiens GN=PINX1 PE=1 SV=2 - [PINX1_HUMAN]	25.2	40.7	38.2	0.0	34.5	0.0
P62841	40S ribosomal protein S15 OS=Homo sapiens GN=RPS15 PE=1 SV=2 - [RS15_HUMAN]	62.5	108.9	39.3	0.0	33.6	0.0

Q8NFH3	Nucleoporin Nup43 OS=Homo sapiens GN=NUP43 PE=1 SV=1 - [NUP43_HUMAN]	79.3	29.7	110.5	0.0	32.1	0.0
O75817	Ribonuclease P protein subunit p20 OS=Homo sapiens GN=POP7 PE=1 SV=2 - [POP7_HUMAN]	67.2	62.5	83.3	0.0	32.0	0.0
P60228	Eukaryotic translation initiation factor 3 subunit E OS=Homo sapiens GN=EIF3E PE=1 SV=1 - [EIF3E_HUMAN]	231.0	304.8	210.2	0.0	30.2	0.0
Q92785	Zinc finger protein ubi-d4 OS=Homo sapiens GN=DPF2 PE=1 SV=2 - [REQU_HUMAN]	62.7	126.8	156.5	0.0	28.9	0.0
Q9BX40	Protein LSM14 homolog B OS=Homo sapiens GN=LSM14B PE=1 SV=1 - [LS14B_HUMAN]	20.4	44.7	56.8	0.0	27.7	0.0
Q9Y3T9	Nucleolar complex protein 2 homolog OS=Homo sapiens GN=NOC2L PE=1 SV=4 - [NOC2L_HUMAN]	71.1	70.0	85.6	0.0	25.8	0.0
P49736	DNA replication licensing factor MCM2 OS=Homo sapiens GN=MCM2 PE=1 SV=4 - [MCM2_HUMAN]	53.8	64.2	54.0	0.0	25.7	0.0
Q8WUM0	Nuclear pore complex protein Nup133 OS=Homo sapiens GN=NUP133 PE=1 SV=2 - [NU133_HUMAN]	343.3	368.1	146.5	0.0	25.6	0.0
Q08J23	tRNA (cytosine(34)-C(5))-methyltransferase OS=Homo sapiens GN=NSUN2 PE=1 SV=2 - [NSUN2_HUMAN]	168.1	118.1	61.6	0.0	21.8	0.0
Q6ZMT4	Lysine-specific demethylase 7A OS=Homo sapiens GN=KDM7A PE=1 SV=2 - [KDM7A_HUMAN]	1678.6	1955.0	1401.1	0.0	0.0	129.5
Q14683	Structural maintenance of chromosomes protein 1A OS=Homo sapiens GN=SMC1A PE=1 SV=2 - [SMC1A_HUMAN]	32.0	96.5	43.7	0.0	0.0	72.0
Q9Y5B9	FACT complex subunit SPT16 OS=Homo sapiens GN=SUPT16H PE=1 SV=1 - [SP16H_HUMAN]	77.2	163.4	110.6	0.0	0.0	50.4
Q9UQE7	Structural maintenance of chromosomes protein 3 OS=Homo sapiens GN=SMC3 PE=1 SV=2 - [SMC3_HUMAN]	118.5	190.6	38.2	0.0	0.0	43.3
Q08945	FACT complex subunit SSRP1 OS=Homo sapiens GN=SSRP1 PE=1 SV=1 - [SSRP1_HUMAN]	345.5	342.2	211.4	0.0	0.0	37.4
Q9UBQ5	Eukaryotic translation initiation factor 3 subunit K OS=Homo sapiens GN=EIF3K PE=1 SV=1 - [EIF3K_HUMAN]	64.4	100.3	57.8	0.0	0.0	36.1
P63244	Guanine nucleotide-binding protein subunit beta-2-like 1 OS=Homo sapiens GN=GNB2L1 PE=1 SV=3 - [GBLP_HUMAN]	381.0	321.9	206.5	0.0	0.0	32.6
Q8NI27	THO complex subunit 2 OS=Homo sapiens GN=THOC2 PE=1 SV=2 - [THOC2_HUMAN]	88.0	104.1	87.4	0.0	0.0	32.3
Q9ULX6	A-kinase anchor protein 8-like OS=Homo sapiens GN=AKAP8L PE=1 SV=3 - [AKP8L_HUMAN]	53.1	61.6	38.4	0.0	0.0	31.8

Q14331	Protein FRG1 OS=Homo sapiens GN=FRG1 PE=1 SV=1 - [FRG1_HUMAN]	49.6	38.6	42.7	0.0	0.0	29.1
Q13601	KRR1 small subunit processome component homolog OS=Homo sapiens GN=KRR1 PE=1 SV=4 - [KRR1_HUMAN]	55.7	35.4	66.4	0.0	0.0	26.4
Q9GZS1	DNA-directed RNA polymerase I subunit RPA49 OS=Homo sapiens GN=POLR1E PE=1 SV=2 - [RPA49_HUMAN]	123.9	132.1	96.9	0.0	0.0	25.8
P78549	Endonuclease III-like protein 1 OS=Homo sapiens GN=NTHL1 PE=1 SV=2 - [NTHL1_HUMAN]	113.0	97.2	44.0	0.0	0.0	23.9
Q6SPF0	Atherin OS=Homo sapiens GN=SAMD1 PE=1 SV=1 - [SAMD1_HUMAN]	30.6	41.4	61.5	0.0	0.0	20.3
O14744	Protein arginine N-methyltransferase 5 OS=Homo sapiens GN=PRMT5 PE=1 SV=4 - [ANM5_HUMAN]	21.5	75.7	24.7	0.0	0.0	0.0

Table S2. Proteome of endogenous KDM7A in Hela cells under 0% FBS condition

Accession	Description	KDM7A (iBAQ)			IgG (iBAQ)		
		Rep 1	Rep 2	Rep 3	Rep 1	Rep 2	Rep 3
P10412	H14_HUMAN Histone H1.4	12981000	25846000	14598000	0	0	0
Q99878	H2A1J_HUMAN Histone H2A type 1-J	11121000	897460	0	0	0	0
Q92686	NEUG_HUMAN Neurogranin	10055000	10260000	0	0	0	0
P62987	RL40_HUMAN Ubiquitin-60S ribosomal protein L40	9890700	15897000	20985000	3995800	0	0
P07910	HNRPC_HUMAN Heterogeneous nuclear ribonucleoproteins C1/C2	7002700	12954000	10341000	0	0	0
Q96PK6	RBM14_HUMAN RNA-binding protein 14	5848100	6722400	5850000	157110	0	0
Q99880	H2B1L_HUMAN Histone H2B type 1-L	5511000	2190900	2606700	5764300	0	0
P09651	ROA1_HUMAN Heterogeneous nuclear ribonucleoprotein A1	5161500	13559000	9240700	0	0	0
P35579	MYH9_HUMAN Myosin-9	5094400	10264000	6130600	0	0	0
P38159	RBMX_HUMAN RNA-binding motif protein, X chromosome	4931600	7996700	5255500	0	0	0
O75367	H2AY_HUMAN Core histone macro-H2A.1	4568900	17588000	5852000	0	0	0
P07305	H10_HUMAN Histone H1.0	4558500	9918600	26612000	0	0	0
P02545	LMNA_HUMAN Prelamin-A/C	3865300	5679300	2757800	0	62354	0
P52272	HNRPM_HUMAN Heterogeneous nuclear ribonucleoprotein M	3738600	4523400	2154400	0	0	0
P60842	IF4A1_HUMAN Eukaryotic initiation factor 4A-I	3321300	4577500	2819600	0	0	0
Q00839	HNRPU_HUMAN Heterogeneous nuclear ribonucleoprotein U	3210400	10499000	7112200	328200	0	0
P06748	NPM_HUMAN Nucleophosmin	3069600	0	2902900	238410	0	0
P22626	ROA2_HUMAN Heterogeneous nuclear ribonucleoproteins A2/B1	2632500	8189500	3749500	0	0	0
P22087	FBRL_HUMAN rRNA 2-O-methyltransferase fibrillarin	2572300	3485000	2447900	0	0	0
P05141	ADT2_HUMAN ADP/ATP translocase 2	2528600	3051900	2929900	0	0	0
Q92522	H1X_HUMAN Histone H1x	2353400	7508800	5010200	0	0	0
P52597	HNRPF_HUMAN Heterogeneous nuclear ribonucleoprotein F	2316800	2876000	4900700	0	0	0
P81605	DCD_HUMAN Dermcidin	2131900	0	1191600	28171000	0	0
Q08211	DHX9_HUMAN ATP-dependent RNA helicase A	1851100	2681100	1838700	0	0	0
P17844	DDX5_HUMAN Probable ATP-dependent RNA helicase DDX5	1746200	1049600	1283300	0	0	0
P11021	BIP_HUMAN Endoplasmic reticulum	1682100	2940000	2123600	0	0	0

	chaperone BiP						
Q9NR30	DDX21_HUMAN Nucleolar RNA helicase 2	1669200	3146200	2148900	0	0	0
P46087	NOP2_HUMAN Probable 28S rRNA (cytosine(4447)-C(5))-methyltransferase	1652900	2586300	2161600	0	0	0
P09874	PARP1_HUMAN Poly [ADP-ribose] polymerase 1	1620600	4164300	3075700	0	0	0
P08238	HS90B_HUMAN Heat shock protein HSP 90-beta	1495100	2108400	783220	0	0	0
Q12905	ILF2_HUMAN Interleukin enhancer-binding factor 2	1278800	2057500	1163500	0	0	0
P06493	CDK1_HUMAN Cyclin-dependent kinase 1	1253800	1227100	1182800	0	0	0
O94875	SRBS2_HUMAN Sorbin and SH3 domain-containing protein 2	1232400	1767400	1078900	0	0	0
Q14980	NUMA1_HUMAN Nuclear mitotic apparatus protein 1	1146700	2290100	1056400	0	0	0
P12814	ACTN1_HUMAN Alpha-actinin-1	1066100	1868900	956870	0	0	0
P55795	HNRH2_HUMAN Heterogeneous nuclear ribonucleoprotein H2	1038300	1841300	1158100	0	0	0
O00571	DDX3X_HUMAN ATP-dependent RNA helicase DDX3X	1034000	2184500	685990	53961	0	0
P61978	HNRPK_HUMAN Heterogeneous nuclear ribonucleoprotein K	987300	1308200	1064800	0	87690	0
P56537	IF6_HUMAN Eukaryotic translation initiation factor 6	964350	1538900	666790	0	0	0
P51659	DHB4_HUMAN Peroxisomal multifunctional enzyme type 2	963630	722320	813210	0	0	0
O00567	NOP56_HUMAN Nucleolar protein 56	872250	2046800	727370	0	0	0
O43795	MYO1B_HUMAN Unconventional myosin-Ib	855070	1599600	942190	0	0	0
P26373	RL13_HUMAN 60S ribosomal protein L13	851040	464890	508890	0	0	0
P11388	TOP2A_HUMAN DNA topoisomerase 2-alpha	837090	1206800	939260	0	0	0
P35580	MYH10_HUMAN Myosin-10	826690	2511700	756860	0	0	0
P04406	G3P_HUMAN Glyceraldehyde-3-phosphate dehydrogenase	809270	5008400	745850	1654400	0	0
P62917	RL8_HUMAN 60S ribosomal protein L8	804450	2850700	1108700	0	0	0
P62701	RS4X_HUMAN 40S ribosomal protein S4, X isoform	789750	1503100	912730	0	0	0
P19338	NUCL_HUMAN Nucleolin	756760	1246900	721610	0	0	0
Q9UHB6	LIMA1_HUMAN LIM domain and actin-binding protein 1	745710	590600	428520	0	0	0
Q8IVT2	MISP_HUMAN Mitotic interactor and	738490	601530	324070	0	0	0

	substrate of PLK1						
P38646	GRP75_HUMAN Stress-70 protein, mitochondrial	737090	792390	313880	62317	0	0
Q99848	EBP2_HUMAN Probable rRNA-processing protein EBP2	734100	488260	1046400	0	0	0
P47755	CAZA2_HUMAN F-actin-capping protein subunit alpha-2	697960	471470	90748	0	0	0
Q15717	ELAV1_HUMAN ELAV-like protein 1	682340	1084300	533820	0	0	0
Q5SSJ5	HP1B3_HUMAN Heterochromatin protein 1-binding protein 3	658280	1231700	892030	0	0	0
P0DMV9	HS71B_HUMAN Heat shock 70 kDa protein 1B	634460	641230	525760	0	0	0
P14625	ENPL_HUMAN Endoplasmin	624250	2330200	878970	0	0	0
Q16891	MIC60_HUMAN MICOS complex subunit MIC60	620730	1015200	591350	0	0	0
P0CW18	PRS56_HUMAN Serine protease 56	597440	1147100	959020	0	0	0
P12268	IMDH2_HUMAN Inosine-5-monophosphate dehydrogenase 2	593720	961400	627150	40603	0	0
Q99959	PKP2_HUMAN Plakophilin-2	593550	1138100	598210	0	0	0
P08254	MMP3_HUMAN Stromelysin-1	590910	0	1174400	0	0	2230500
P14866	HNRPL_HUMAN Heterogeneous nuclear ribonucleoprotein L	586160	1137100	229110	0	0	0
O95425	SVIL_HUMAN Supervillin	582240	689260	514740	0	0	0
P13639	EF2_HUMAN Elongation factor 2	521000	1215000	622900	42152	0	0
Q86V81	THOC4_HUMAN THO complex subunit 4	515590	2007200	1047900	0	0	0
Q13813	SPTN1_HUMAN Spectrin alpha chain, non-erythrocytic 1	510720	642220	533130	0	0	0
Q12906	ILF3_HUMAN Interleukin enhancer-binding factor 3	502400	1450200	515350	0	0	0
P46439	GSTM5_HUMAN Glutathione S-transferase Mu 5	494670	792390	548480	0	0	0
O43175	SERA_HUMAN D-3-phosphoglycerate dehydrogenase	493030	269050	122910	0	0	0
P62753	RS6_HUMAN 40S ribosomal protein S6	473720	1290900	444990	0	0	0
Q01082	SPTB2_HUMAN Spectrin beta chain, non-erythrocytic 1	465340	527650	464960	0	0	0
P62140	PP1B_HUMAN Serine/threonine-protein phosphatase PP1-beta catalytic subunit	448060	771030	446180	0	0	0
P52907	CAZA1_HUMAN F-actin-capping protein subunit alpha-1	443400	944790	562000	0	0	0
Q9H6F5	CCD86_HUMAN Coiled-coil domain-containing protein 86	440520	3090600	1625500	0	0	0

Q15366	PCBP2_HUMAN Poly(rC)-binding protein 2	435350	1565200	534450	0	0	0
P11387	TOP1_HUMAN DNA topoisomerase 1	428420	937900	441790	0	0	0
P67809	YBOX1_HUMAN Nuclease-sensitive element-binding protein 1	424210	673190	208140	0	0	0
P10809	CH60_HUMAN 60 kDa heat shock protein, mitochondrial	419180	618070	454140	0	0	0
P51991	ROA3_HUMAN Heterogeneous nuclear ribonucleoprotein A3	408390	2778100	2101500	0	0	0
P43243	MATR3_HUMAN Matrin-3	400970	1326800	899710	0	0	0
P46013	KI67_HUMAN Proliferation marker protein Ki-67	390170	620150	608040	0	0	0
Q9NVI7	ATD3A_HUMAN ATPase family AAA domain-containing protein 3A	384250	966670	739470	0	0	0
P38919	IF4A3_HUMAN Eukaryotic initiation factor 4A-III	375300	427610	443050	0	0	0
Q07955	SRSF1_HUMAN Serine/arginine-rich splicing factor 1	370500	736780	584720	0	0	0
P14923	PLAK_HUMAN Junction plakoglobin	366990	689140	437010	729120	0	0
Q8TDN6	BRX1_HUMAN Ribosome biogenesis protein BRX1 homolog	352670	750450	467240	0	0	0
O95816	BAG2_HUMAN BAG family molecular chaperone regulator 2	350300	1150700	994760	0	0	0
P49327	FAS_HUMAN Fatty acid synthase	348800	490960	356980	0	0	0
Q14126	DSG2_HUMAN Desmoglein-2	339700	446200	222550	0	0	0
Q99714	HCD2_HUMAN 3-hydroxyacyl-CoA dehydrogenase type-2	323800	278710	0	0	0	0
Q9Y2Q3	GSTK1_HUMAN Glutathione S-transferase kappa 1	321720	432830	412170	0	0	0
Q15233	NONO_HUMAN Non-POU domain-containing octamer-binding protein	308150	701930	833170	0	0	0
P39023	RL3_HUMAN 60S ribosomal protein L3	303580	551740	347760	0	0	0
Q96HS1	PGAM5_HUMAN Serine/threonine-protein phosphatase PGAM5, mitochondrial	294760	598400	287740	0	0	0
Q9H0A0	NAT10_HUMAN RNA cytidine acetyltransferase	290930	414670	181750	0	0	0
P33993	MCM7_HUMAN DNA replication licensing factor MCM7	285510	444920	112960	0	0	0
P42704	LPPRC_HUMAN Leucine-rich PPR motif-containing protein, mitochondrial	276800	285700	308670	0	0	0
O43143	DHX15_HUMAN Pre-mRNA-splicing factor ATP-dependent RNA helicase DHX15	276420	764680	182600	0	0	0

Q8N3V7	SYNPO_HUMAN Synaptopodin	274180	654250	267410	0	0	0
Q9NX63	MIC19_HUMAN MICOS complex subunit MIC19	272850	1678400	292260	0	0	0
P14618	KPYM_HUMAN Pyruvate kinase PKM	267910	480670	260510	0	0	0
Q9BY77	PDIP3_HUMAN Polymerase delta-interacting protein 3	265270	591620	93358	0	0	0
P63244	RACK1_HUMAN Receptor of activated protein C kinase 1	256680	411430	119620	0	0	0
Q16629	SRSF7_HUMAN Serine/arginine-rich splicing factor 7	248160	341030	288400	0	0	0
P12956	XRCC6_HUMAN X-ray repair cross-complementing protein 6	237240	479370	266730	0	0	0
P35659	DEK_HUMAN Protein DEK	230500	1419600	258250	0	0	0
Q8WXI9	P66B_HUMAN Transcriptional repressor p66-beta	226790	120590	190040	0	0	0
Q14974	IMB1_HUMAN Importin subunit beta-1	226490	257790	0	0	0	0
P11586	C1TC_HUMAN C-1-tetrahydrofolate synthase, cytoplasmic	225720	390040	229830	0	0	0
Q9UN86	G3BP2_HUMAN Ras GTPase-activating protein-binding protein 2	225070	231610	0	0	0	0
Q9BQG0	MBB1A_HUMAN Myb-binding protein 1A	215310	245560	315860	0	0	0
P40938	RFC3_HUMAN Replication factor C subunit 3	213400	255320	266360	0	0	0
P43405	KSYK_HUMAN Tyrosine-protein kinase SYK	211470	0	344830	0	0	0
Q6YN16	HSDL2_HUMAN Hydroxysteroid dehydrogenase-like protein 2	209800	414480	190640	0	0	0
Q9UQ35	SRRM2_HUMAN Serine/arginine repetitive matrix protein 2	204580	324720	196070	0	0	0
P31689	DNJA1_HUMAN DnaJ homolog subfamily A member 1	204150	228420	201070	0	0	0
P21333	FLNA_HUMAN Filamin-A	199990	257820	210820	0	0	0
Q14103	HNRPD_HUMAN Heterogeneous nuclear ribonucleoprotein D0	198000	378980	281940	0	0	0
Q02878	RL6_HUMAN 60S ribosomal protein L6	195910	674170	390550	0	0	0
Q9NYL9	TMOD3_HUMAN Tropomodulin-3	189570	495740	351810	0	0	0
O60264	SMCA5_HUMAN SWI/SNF-related matrix-associated actin-dependent regulator of chromatin subfamily A member 5	188380	325680	193290	0	0	0
O43390	HNRPR_HUMAN Heterogeneous nuclear ribonucleoprotein R	169800	350720	408300	0	0	0
P35249	RFC4_HUMAN Replication factor C subunit	165780	1014700	320010	0	0	0

	4						
Q14244	MAP7_HUMAN Ensconsin	163840	839550	328670	0	0	0
P62995	TRA2B_HUMAN Transformer-2 protein homolog beta	161560	310360	0	0	0	0
Q15645	PCH2_HUMAN Pachytene checkpoint protein 2 homolog	160470	515240	0	0	0	0
P56182	RRP1_HUMAN Ribosomal RNA processing protein 1 homolog A	159190	115810	145080	0	0	0
P18124	RL7_HUMAN 60S ribosomal protein L7	157330	330090	302730	0	0	0
P35250	RFC2_HUMAN Replication factor C subunit 2	157000	201230	340140	0	0	0
P34897	GLYM_HUMAN Serine hydroxymethyltransferase, mitochondrial	152110	124930	300600	0	0	0
Q9NVP1	DDX18_HUMAN ATP-dependent RNA helicase DDX18	146560	419890	273150	0	0	0
O15213	WDR46_HUMAN WD repeat-containing protein 46	145040	72416	0	0	0	0
P11498	PYC_HUMAN Pyruvate carboxylase, mitochondrial	143830	277740	178210	0	0	0
Q9H307	PININ_HUMAN Pinin	139290	262970	0	0	0	0
Q07666	KHDR1_HUMAN KH domain-containing, RNA-binding, signal transduction-associated protein 1	139080	0	223100	0	0	0
Q9UJZ1	STML2_HUMAN Stomatin-like protein 2, mitochondrial	138130	178460	106580	0	0	0
P23396	RS3_HUMAN 40S ribosomal protein S3	136980	396430	198570	0	0	0
O75390	CISY_HUMAN Citrate synthase, mitochondrial	136520	614880	236830	0	0	0
P18887	XRCC1_HUMAN DNA repair protein XRCC1	130790	220870	147310	0	0	0
P42166	LAP2A_HUMAN Lamina-associated polypeptide 2, isoform alpha	130250	476500	158050	0	0	0
P49790	NU153_HUMAN Nuclear pore complex protein Nup153	130100	150480	52522	0	0	0
Q99459	CDC5L_HUMAN Cell division cycle 5-like protein	124350	86884	0	0	0	0
O43865	SAHH2_HUMAN S-adenosylhomocysteine hydrolase-like protein 1	123420	215790	136650	0	0	0
O00116	ADAS_HUMAN Alkyldihydroxyacetonephosphate synthase, peroxisomal	122230	80850	68424	0	0	0
P33992	MCM5_HUMAN DNA replication licensing factor MCM5	117410	149640	67449	0	0	0

Q14684	RRP1B_HUMAN Ribosomal RNA processing protein 1 homolog B	116310	290610	0	0	0	0
O43707	ACTN4_HUMAN Alpha-actinin-4	114120	215530	93161	0	0	0
O00303	EIF3F_HUMAN Eukaryotic translation initiation factor 3 subunit F	112890	150140	103590	0	0	0
P47756	CAPZB_HUMAN F-actin-capping protein subunit beta	112390	1348100	339650	0	0	0
P04843	RPN1_HUMAN Dolichyl-diphosphooligosaccharide--protein glycosyltransferase subunit 1	111220	139560	138570	0	0	0
Q16643	DREB_HUMAN Drebrin	110360	225880	170400	0	0	0
P49916	DNLI3_HUMAN DNA ligase 3	103240	203300	123560	0	0	0
P51648	AL3A2_HUMAN Fatty aldehyde dehydrogenase	102010	173020	0	0	0	0
P55265	DSRAD_HUMAN Double-stranded RNA-specific adenosine deaminase	101200	123010	37017	0	0	0
P11940	PABP1_HUMAN Polyadenylate-binding protein 1	99111	267240	156480	0	0	0
Q9BVP2	GNL3_HUMAN Guanine nucleotide-binding protein-like 3	98873	503800	365000	0	0	0
Q15269	PWP2_HUMAN Periodic tryptophan protein 2 homolog	98123	70076	0	0	0	0
Q9H0S4	DDX47_HUMAN Probable ATP-dependent RNA helicase DDX47	97389	163290	132120	0	0	0
Q8TEM1	PO210_HUMAN Nuclear pore membrane glycoprotein 210	95299	271420	69850	0	0	0
Q8TDM6	DLG5_HUMAN Disks large homolog 5	94470	130610	46036	0	0	0
P26599	PTBP1_HUMAN Polypyrimidine tract-binding protein 1	93769	112360	90306	0	0	0
P07199	CENPB_HUMAN Major centromere autoantigen B	92902	61710	0	0	0	0
Q14697	GANAB_HUMAN Neutral alpha-glucosidase AB	90901	316260	0	0	0	0
Q71RC2	LARP4_HUMAN La-related protein 4	87611	136530	121450	0	0	0
P46940	IQGA1_HUMAN Ras GTPase-activating-like protein IQGAP1	86227	318190	108130	0	0	0
Q9Y2W1	TR150_HUMAN Thyroid hormone receptor-associated protein 3	85653	284680	101700	0	0	0
Q96JB3	HIC2_HUMAN Hypermethylated in cancer 2 protein	83346	146180	0	0	0	0
O60568	PLOD3_HUMAN Multifunctional procollagen lysine hydroxylase and glycosyltransferase LH3	83025	115080	70712	0	0	0

O14974	MYPT1_HUMAN Protein phosphatase 1 regulatory subunit 12A	82921	287640	96846	0	0	0
Q9H0D6	XRN2_HUMAN 5-3 exoribonuclease 2	81020	326420	152390	0	0	0
P31153	METK2_HUMAN S-adenosylmethionine synthase isoform type-2	80734	219310	114850	0	0	0
P25205	MCM3_HUMAN DNA replication licensing factor MCM3	80495	221350	0	0	0	0
P35251	RFC1_HUMAN Replication factor C subunit 1	78829	64915	115290	0	0	0
P23921	RIR1_HUMAN Ribonucleoside-diphosphate reductase large subunit	75753	230970	99336	0	0	0
P07900	HS90A_HUMAN Heat shock protein HSP 90-alpha	72216	86787	0	0	0	0
Q7KZF4	SND1_HUMAN Staphylococcal nuclease domain-containing protein 1	71003	142990	67186	0	0	0
Q13435	SF3B2_HUMAN Splicing factor 3B subunit 2	68809	181070	36259	0	0	0
Q00610	CLH1_HUMAN Clathrin heavy chain 1	64733	78314	52786	0	0	0
Q9BZE4	NOG1_HUMAN Nucleolar GTP-binding protein 1	64486	650720	513370	0	0	0
Q12788	TBL3_HUMAN Transducin beta-like protein 3	64293	52795	28418	0	0	0
P01024	CO3_HUMAN Complement C3	63547	50313	0	0	0	0
Q9UIG0	BAZ1B_HUMAN Tyrosine-protein kinase BAZ1B	63203	135840	69042	0	0	0
Q8NI36	WDR36_HUMAN WD repeat-containing protein 36	61670	74340	23932	0	0	0
P51116	FXR2_HUMAN Fragile X mental retardation syndrome-related protein 2	61085	114380	221660	0	0	0
Q15029	U5S1_HUMAN 116 kDa U5 small nuclear ribonucleoprotein component	59485	158450	48841	0	0	0
Q99590	SCAFB_HUMAN Protein SCAF11	59241	34216	0	0	0	0
Q96GQ7	DDX27_HUMAN Probable ATP-dependent RNA helicase DDX27	58516	195860	127700	0	0	0
P56192	SYMC_HUMAN Methionine--tRNA ligase, cytoplasmic	58047	129100	110550	0	0	0
Q9NW13	RBM28_HUMAN RNA-binding protein 28	54405	271460	78016	0	0	0
P50991	TCPD_HUMAN T-complex protein 1 subunit delta	54183	120290	65922	0	0	0
Q9Y5B9	SP16H_HUMAN FACT complex subunit SPT16	53644	227030	69410	0	0	0
Q8WYP5	ELYS_HUMAN Protein ELYS	52507	192690	50772	0	0	0
Q14966	ZN638_HUMAN Zinc finger protein 638	49804	81488	53384	0	0	0

P17858	PFKAL_HUMAN ATP-dependent 6-phosphofructokinase, liver type	48961	320260	0	0	0	0
Q6ZRV2	FA83H_HUMAN Protein FAM83H	45629	159800	55504	0	0	0
P54886	P5CS_HUMAN Delta-1-pyrroline-5-carboxylate synthase	41280	29062	40468	0	0	0
Q9NY93	DDX56_HUMAN Probable ATP-dependent RNA helicase DDX56	40986	106670	0	0	0	0
Q15154	PCM1_HUMAN Pericentriolar material 1 protein	30847	34203	0	0	0	0
Q99832	TCPH_HUMAN T-complex protein 1 subunit eta	30527	23387	86933	0	0	0
Q14690	RRP5_HUMAN Protein RRP5 homolog	29058	161880	0	0	0	0
Q13085	ACACA_HUMAN Acetyl-CoA carboxylase 1	25670	10796	9743	0	0	0
Q02880	TOP2B_HUMAN DNA topoisomerase 2-beta	24144	227530	92293	0	0	0
Q7Z2W4	ZCCHV_HUMAN Zinc finger CCCH-type antiviral protein 1	24098	0	45848	0	0	0
P48681	NEST_HUMAN Nestin	23513	57119	0	0	0	0
O75369	FLNB_HUMAN Filamin-B	23393	41285	16552	0	0	0
Q9H8H2	DDX31_HUMAN Probable ATP-dependent RNA helicase DDX31	21215	0	22707	0	0	0
P58107	EPIPL_HUMAN Epiplakin	20125	96022	42310	0	0	0
Q7L2E3	DHX30_HUMAN ATP-dependent RNA helicase DHX30	19607	108830	20453	0	0	0
P49750	YLPM1_HUMAN YLP motif-containing protein 1	18460	13947	0	0	0	0
Q8WWI1	LMO7_HUMAN LIM domain only protein 7	18380	89939	62435	0	0	0
Q14204	DYHC1_HUMAN Cytoplasmic dynein 1 heavy chain 1	7867.2	21154	0	0	0	0
O14639	ABLM1_HUMAN Actin-binding LIM protein 1	0	87604	61315	0	0	0
O43159	RRP8_HUMAN Ribosomal RNA-processing protein 8	0	89673	87899	0	0	0
O76021	RL1D1_HUMAN Ribosomal L1 domain-containing protein 1	0	499410	74041	0	0	0
P04181	OAT_HUMAN Ornithine aminotransferase, mitochondrial	0	222450	172740	0	0	0
P04792	HSPB1_HUMAN Heat shock protein beta-1	0	265010	121090	339220	0	0
P06576	ATPB_HUMAN ATP synthase subunit beta, mitochondrial	0	202150	129200	248930	0	0
P08559	ODPA_HUMAN Pyruvate dehydrogenase E1 component subunit alpha, somatic form, mitochondrial	0	107760	143110	0	0	0

P17066	HSP76_HUMAN Heat shock 70 kDa protein 6	0	364120	296650	0	0	0
P22314	UBA1_HUMAN Ubiquitin-like modifier-activating enzyme 1	0	71597	63373	0	0	0
P27635	RL10_HUMAN 60S ribosomal protein L10	0	1844800	1239400	0	0	0
P35998	PRS7_HUMAN 26S proteasome regulatory subunit 7	0	85337	29544	0	0	0
P40937	RFC5_HUMAN Replication factor C subunit 5	0	320380	90758	0	0	0
P45880	VDAC2_HUMAN Voltage-dependent anion-selective channel protein 2	0	765380	580130	0	0	0
P47914	RL29_HUMAN 60S ribosomal protein L29	0	1277200	845290	0	0	0
P50990	TCPQ_HUMAN T-complex protein 1 subunit theta	0	213200	30967	0	0	0
P53396	ACLY_HUMAN ATP-citrate synthase	0	41152	53905	0	0	0
P61353	RL27_HUMAN 60S ribosomal protein L27	0	866760	339230	0	0	0
P62191	PRS4_HUMAN 26S proteasome regulatory subunit 4	0	125130	102430	0	0	0
P62241	RS8_HUMAN 40S ribosomal protein S8	0	1448200	467970	0	0	0
P62424	RL7A_HUMAN 60S ribosomal protein L7a	0	2727300	1143600	0	0	0
P62906	RL10A_HUMAN 60S ribosomal protein L10a	0	4657200	1836200	0	0	0
P78347	GTF2I_HUMAN General transcription factor II-I	0	151350	75647	0	0	0
P78527	PRKDC_HUMAN DNA-dependent protein kinase catalytic subunit	0	27283	20556	0	0	0
Q02978	M2OM_HUMAN Mitochondrial 2-oxoglutarate/malate carrier protein	0	574920	485760	0	0	0
Q06210	GFPT1_HUMAN Glutamine--fructose-6-phosphate aminotransferase [isomerizing] 1	0	25089	23821	0	0	0
Q13148	TADBP_HUMAN TAR DNA-binding protein 43	0	541700	276590	0	0	0
Q13200	PSMD2_HUMAN 26S proteasome non-ATPase regulatory subunit 2	0	86475	59336	0	0	0
Q13247	SRSF6_HUMAN Serine/arginine-rich splicing factor 6	0	248690	100050	0	0	0
Q13547	HDAC1_HUMAN Histone deacetylase 1	0	224350	166730	0	0	0
Q14498	RBM39_HUMAN RNA-binding protein 39	0	65048	62714	59025	0	0
Q15365	PCBP1_HUMAN Poly(rC)-binding protein 1	0	796460	522630	0	0	0
Q15393	SF3B3_HUMAN Splicing factor 3B subunit 3	0	128520	37819	0	0	0

Q15424	SAFB1_HUMAN Scaffold attachment factor B1	0	182050	0	0	0	0
Q15517	CDSN_HUMAN Corneodesmosin	0	0	0	725290	0	0
Q49A26	GLYR1_HUMAN Putative oxidoreductase GLYR1	0	50906	0	0	0	0
Q562R1	ACTBL_HUMAN Beta-actin-like protein 2	0	0	69910000	0	0	0
Q5BKZ1	ZN326_HUMAN DBIRD complex subunit ZNF326	0	132860	0	0	0	0
Q5T280	CI114_HUMAN Putative methyltransferase C9orf114	0	107210	0	0	0	0
Q5T3J3	LRIF1_HUMAN Ligand-dependent nuclear receptor-interacting factor 1	0	380350	0	0	0	0
Q6GYQ0	RGPA1_HUMAN Ral GTPase-activating protein subunit alpha-1	0	0	66439	0	0	0
Q6PL18	ATAD2_HUMAN ATPase family AAA domain-containing protein 2	0	84876	30809	0	0	0
Q6UWP8	SBSN_HUMAN Suprabasin	0	0	0	1609800	0	0
Q6WCQ1	MPRIP_HUMAN Myosin phosphatase Rho-interacting protein	0	228010	47116	0	0	0
Q7Z7K6	CENPV_HUMAN Centromere protein V	0	471380	505360	0	0	0
Q86U42	PABP2_HUMAN Polyadenylate-binding protein 2	0	353980	110180	0	0	0
Q86V48	LUZP1_HUMAN Leucine zipper protein 1	0	0	73244	0	0	0
Q86XI8	ZSWM9_HUMAN Uncharacterized protein ZSWIM9	0	0	0	198290	0	0
Q8IZL8	PELP1_HUMAN Proline-, glutamic acid- and leucine-rich protein 1	0	130090	0	0	0	0
Q8N556	AFAP1_HUMAN Actin filament-associated protein 1	0	0	209440	0	0	0
Q8NDH6	ICA1L_HUMAN Islet cell autoantigen 1-like protein	0	0	0	0	0	0
Q8TDD1	DDX54_HUMAN ATP-dependent RNA helicase DDX54	0	186170	102270	0	0	0
Q92616	GCN1_HUMAN eIF-2-alpha kinase activator GCN1	0	67879	11830	0	0	0
Q99729	ROAA_HUMAN Heterogeneous nuclear ribonucleoprotein A/B	0	441790	316100	0	0	0
Q9H7B2	RPF2_HUMAN Ribosome production factor 2 homolog	0	610120	237680	0	0	0
Q9NWH9	SLTM_HUMAN SAFB-like transcription modulator	0	410880	124210	0	0	0
Q9UKM9	RALY_HUMAN RNA-binding protein Raly	0	134690	95658	0	0	0

Q9ULW0	TPX2_HUMAN Targeting protein for Xklp2	0	61976	64138	0	0	0
Q9Y2R4	DDX52_HUMAN Probable ATP-dependent RNA helicase DDX52	0	86060	163340	0	0	0
Q9Y2X3	NOP58_HUMAN Nucleolar protein 58	0	1709200	68555	0	0	0
Q9Y5Q9	TF3C3_HUMAN General transcription factor 3C polypeptide 3	0	48118	27435	0	0	0
